# Determining buffer conditions for downstream processing of VLP-based recombinant hepatitis B surface antigen using multimodal resins in bind-elute and flow-through purification modes

**DOI:** 10.1038/s41598-023-37614-y

**Published:** 2023-07-03

**Authors:** Maryam Moazami Goodarzi, Reza Jalalirad, Delaram Doroud, Hamidreza Hozouri, Mohammadreza Aghasadeghi, Mahdi Paryan

**Affiliations:** 1 Department of Research and Development, Production and Research Complex, Pasteur Institute of Iran, Karaj, 3159915111 Iran; 2 Department of Quality Management, Production and Research Complex, Pasteur Institute of Iran, Karaj, 3159915111 Iran; 3 Department of Hepatitis and AIDS, Pasteur Institute of Iran, Tehran, 1316943551 Iran; 4 Viral Vaccine Research Center, Pasteur Institute of Iran, Tehran, 1316943551 Iran

**Keywords:** Chemical engineering, Biotechnology

## Abstract

The difficulties in purification of VLP-based recombinant hepatitis B surface antigen (rHBsAg) are mainly emerged from inefficient semi-purification step plus proteins physicochemical properties and these issues make the downstream processing (DSP) very lengthy and expensive. In this study, optimization of rHBsAg (recombinantly-expressed in *Pichia pastoris*) DSP was performed using selection of buffering conditions in the semi-purification step. In the semi-purification optimization step, up to 73% of the protein impurities were eliminated and the utmost increase in rHBsAg purity (ca. 3.6-fold) was achieved using 20 mM sodium acetate, pH 4.5. By using rHBsAg binding and nonbinding situations obtained from the response surface plot in design of experiments (DOE), additional bind-elute and flow-through purification mode experiments were conducted and rHBsAg with high purity (near 100%) and recovery (> 83%) was achieved. Following assessment of critical quality attributes (i.e., purity, particle size distribution, host cell DNA, host cell protein, secondary structures, specific activity and relative potency), it was indicated that the characteristics of rHBsAg purified by the new DSP were similar or superior to the ones obtained from conventional DSP. The purification performance of the resin was constantly retained (97–100%) and no significant resin damage took place after 10 adsorption–elution–cleaning cycles. The new DSP developed for production of rHBsAg in this study can substitute the conventional one with granting satisfactory target protein quality, long-lasting resin efficacy, shorter and less expensive process. This process may be also employable for purification of both non-VLP- and VLP- based target proteins expressed in the yeast.

## Introduction

Hepatitis B virus (HBV) accounts for around 80% of the global burden of hepatocellular carcinoma (HCC) and finally 820,000 deaths annually^1–3^. Purification of hepatitis B surface antigen (HBsAg) from the plasma of the virus carriers was the initial common source of HBV vaccines; however, the inadequate supply of human plasma plus increased risk of viral transmission were the main motives to the production of recombinant HBsAg^4–7^. Like other virus-like particles (VLPs), rHBsAg has also the potential to be used as vaccine carrier and gene therapy vector^8–10^.

In the production route of pharmaceutical recombinant proteins, complications of downstream processing (DSP) are yet responsible for the major part of the manufacturing processing cost. Such complications are exacerbated for VLP based proteins as the large size of the particles slows down and even prevents their diffusion through chromatographic resins pores and their access to the large internal surface areas. These issues afflict the resins performance in terms of binding capacity, VLPs recovery and purity^10,11^.

DSP of the therapeutic proteins generally initiates with methods such as clarification by filtration or precipitation, to diminish the load of contaminants, and then it is perpetuated using several expensive chromatography steps to reach a very high purity (near to 100%)^12,13^. Although chromatography is still the key player in the biopharmaceuticals purification, the efficiency of non-chromatographic steps (in removing impurities) has effects on the number of subsequent chromatography steps, the chromatography resins life-time and the overall process economy^14–16^. In the production process of rHBsAg vaccine using *Pichia pastoris* (*P. pastoris)*, mechanical cell disruption and extraction methods are usually used subsequent to the cultivation stage in order to release the yeast cell-derived HBsAg. Following such operations, the complete soluble cell components (i.e., host cell DNA (HCD), host cell protein (HCP) and lipids) are also discharged into the crude cell extract. As a result, a complex purification process (comprised of acid precipitation, aerosile adsorption–desorption, centrifugation, ultrafiltration and purification by sequential immunoaffinity—ion exchange (IEX)—gel filtration (GF) chromatography) is required in order to obtain a highly pure rHBsAg which is used for preparation of active pharmaceutical ingredient (rHBsAg-API) at a large-scale^17^.

In the conventional process of purifying rHBsAg originated from recombinant *P. pastoris*, acid precipitation is performed in the presence of 3 M potassium thiocyanate (KSCN) as an initial protein recovery step (namely semi-purification). The increase in the purity of rHBsAg throughout this semi-purification step is insignificant (ca. up to 5%)^17,18^. KSCN is a chaotropic salt increasing the solubility of the protein by decreasing the surface tension of the solution. Therefore, when acid (or isoelectric) precipitation takes place in the presence of KSCN, many protein impurities do not precipitate at their specific isoelectric points (PIs) and they remain soluble in the solution. This leads to a huge load of protein impurities in the solution and consequently makes rHBsAg purification difficult^19^.

**Figure 1:**
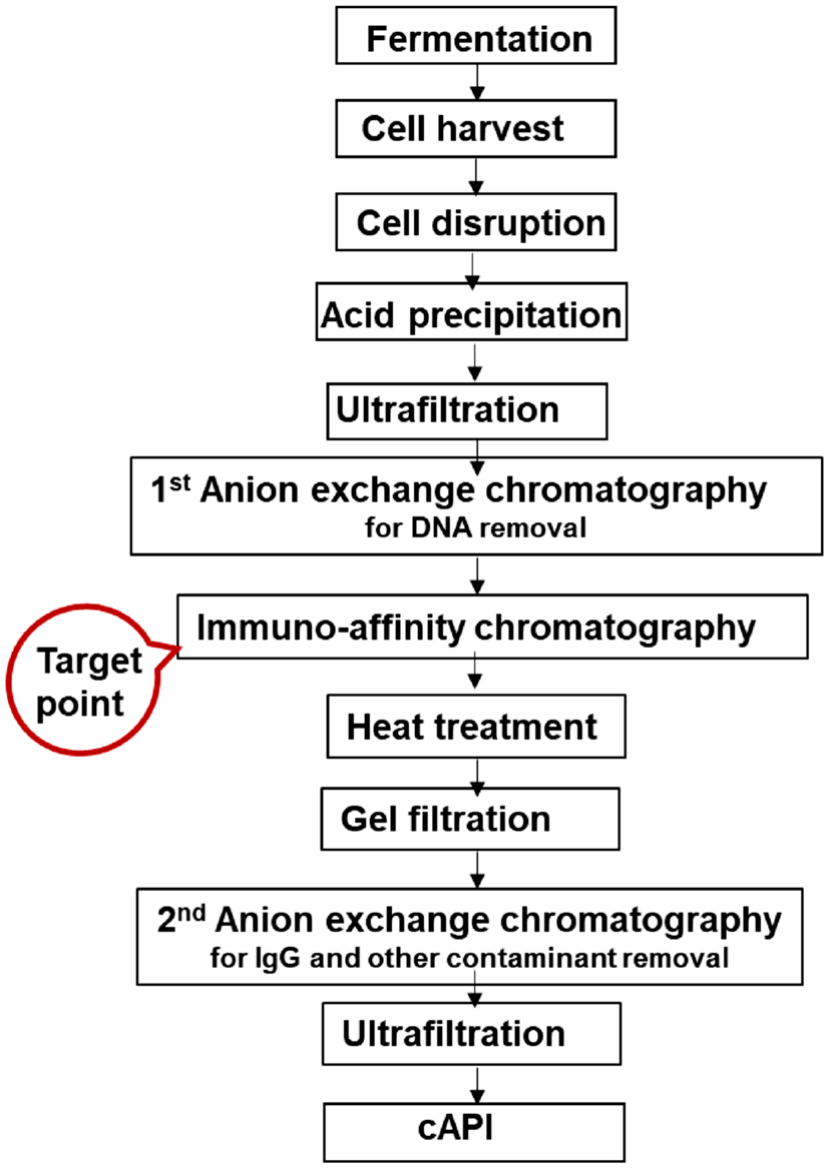
Flowchart for the conventional rHBsAg production process.

In the conventional downstream processing of rHBsAg originated from recombinant *P. pastoris* (Fig. 1), immunoaffinity chromatography plays an integral role, since it effectively removes most of the impurities (i.e., host cell proteins, DNA impurities)^17^. However, the serious drawbacks of the immunoaffinity resin (such as exclusive and complex commercial resin manufacturing^17,20,21^, low resistance of the resin to chemical cleaning^22–24^, ligand leakage which can potentially lead to product contamination with viruses and monoclonal antibodies) makes the downstream process complicated, time-consuming and costly^17,25^. Therefore, it is presumed that by achieving substantial reduction of protein impurities during semi-purification process, purification of rHBsAg (with high purity) using more available, efficient and cost-effective non-affinity chromatography resins (such as multimodal ones) would subsequently become plausible**.** In comparison to the traditional single-mode resins, mixed-mode (multimodal) resins interact with the adsorbates via multiple interactions (i.e., ion exchange, hydrophobic interaction, hydrogen bonds and Wander Waals forces) which increase their adsorption capacity and selectivity^26,27^.

In this study, the effect of removing KSCN from *P. pastoris* feedstock containing rHBsAg on the reduction of protein impurities in semi-purification step was firstly investigated by adjusting various pH values in different buffers. The samples obtained from the most effective semi-purification step were then utilized for further purification of rHBsAg using multimodal Capto MMC resin in bind-elute and flow-through modes. As the complexity of multimodal interactions in such resins makes it difficult to characterize the resin behaviors in the purification process, design space was defined using design of experiments (DOE) before performing bind-elute and flow-through mode experiments with Capto MMC resin. In order to determine product compliance, the quality attributes of the product obtained from the optimized purification process using Capto MMC resin were compared to those of the conventional product. The suitability of Capto MMC resin for replacing burdensome IAF chromatography and other chromatographic purification methods in the conventional downstream processing was determined based on the achieved quality attributes.

## Results

### Investigation of the effect of KSCN removal on the reduction of protein impurities in different buffering conditions

**Figure 2:**
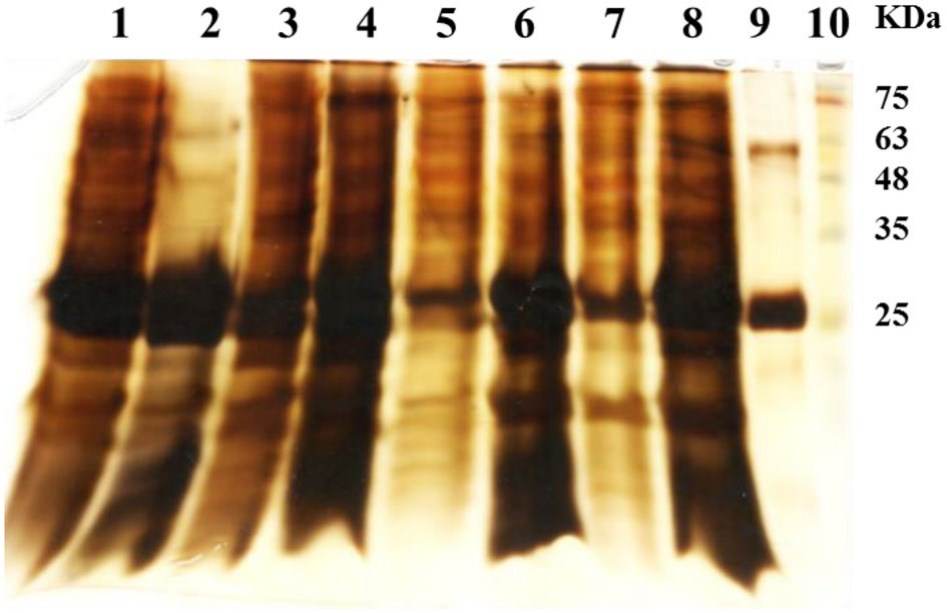
SDS-PAGE analysis of the supernatant sample dialyzed in various buffering conditions. Lanes: 1—The SAP sample, 2 and 3—The supernatant and pellet of the sample dialyzed in 20 mM sodium acetate, pH 4.5, respectively, 4 and 5—The supernatant and pellet of the sample dialyzed in 20 mM Tris–HCl, pH 4.5, respectively, 6 and 7—The supernatant and pellet of the sample dialyzed in 20 mM sodium acetate, pH 5.5, respectively, 8—The supernatant of the sample dialyzed in 20 mM sodium phosphate, pH 6.5, 9—Conventional rHBsAg API (cAPI) and 10– Protein makers.

Following removal of KSCN at pH lower than 7.2, various impurities were precipitated to different extents in varying buffering conditions (Fig. 2).

The results of these experiments showed that about 75% of total protein was precipitated in sodium acetate, pH 4.5. At such condition, 3% of the precipitated proteins was related to rHBsAg (being equivalent to 21% of total rHBsAg existed in the SAP). In Tris–HCl buffer pH 4.5, around 27% of total protein was precipitated and about 12% of rHBsAg was also lost. In sodium acetate, pH 5.5, the amount of total protein precipitation and rHBsAg loss were about 30% and 14%, respectively. Dialysis of the SAP in sodium phosphate (pH 6.5) led to the lowest total protein precipitation and rHBsAg loss (14% and 9%, respectively). The detailed results related to total protein precipitation, rHBsAg recovery and purity in different fractions obtained during these experiments with various buffering conditions have been reported in Table 1.

**Table 1:** The results of decreasing protein impurities in the SAP following dialysis in different buffer conditions.

Buffer type	Total protein in supernatant (µg/ml)	Total protein in pellet (µg/ml)	rHBsAg in supernatant (µg/ml)	rHBsAg in pellet (µg/ml)	rHBsAg recovery* (%)	Step purification fold (PF)**
20 mM Tris–HCl, 3 M KSCN, pH 7.2	1957	0	213	10	100	1
20 mM Sodium acetate, pH 4.5	477	1480	169	35.4	79	3.54
50 mM Tris–HCl, pH 4.5	1424	533	187	13.1	88	1.31
20 mM Sodium acetate, pH 5.5	1377	580	183	13.3	86	1.33
20 mM Sodium phosphate, pH 6.5	1677	280	194	11.5	91	1.15

*(rHBsAg content in supernatant divided by rHBsAg content in SAP) multiplied by 100.

**rHBsAg purity obtained after buffer exchange divided by rHBsAg purity in SAP.

### Defining CQAs, CPPs, and CMAs

With the intention of efficient improvement in industrial rHBsAg downstream processing using Capto MMC resin, the design space elements such as critical quality attributes (CQAs), critical process parameters (CPPs) and critical material attributes (CMA) were defined using the established large-scale rHBsAg quality control in the conventional manufacturing process and information on the subject being available in literature. CQAs of rHBsAg are afflicted by product-related as well as host-related impurities, and they are varied based on process step requirements. For the conventional downstream processing to produce cAPI (Fig. 1)^29^, CQAs for the most important steps have been demonstrated in Table 2.

**Table 2:** Critical quality attributes and analytical tools relevant to some of the most important steps in the conventional rHBsAg downstream processing.

DSP step	CQAs	Analytical tool
Immunoaffinity chromatography	Purity Particle size distribution	SDS-PAGE, SEC-HPLC SEC-HPLC
Ultrafiltration	Purity Particle size distribution Potency Specific activity HCD HCP	SDS-PAGE and SEC-HPLC SEC-HPLC Enzyme immunoassay Enzyme immunoassay qPCR Enzyme immunoassay

According to this information, it is evident that the situation is more demanding as the process moves towards the end of downstream processing, due to the fact that the values of several CQAs should fall into the permissible limits. Salt type/concentration as CMAs, and pH as the most important CPP, were selected for the experiments in this study.

## Defining design space and purification of rHBsAg in bind-elute mode

### Optimization step for rHBsAg binding on Capto MMC resin

The binding optimization was performed based on DOE as effective approach to achieve global optima and robust process. The response surface methodology (RSM) was selected to support main effects, interaction and curvature effects in the protein purification. Amongst RSM designs, composite face-centered (CCF) design as a type of central composite design (CCD) is suitable due to the fact that it helps design experiments in the desired range and supports low variables. These experiments were performed using rHBsAg-API to specifically clarify the behavior of rHBsAg and Capto MMC resin.

The experimental data collected from the execution of the design layouts (containing 22 runs) have been presented in Tables 3a and 3d.

**Table 3:** The design layouts and the face-centered design results for binding rHBsAg on Capto MMC resin in the presence of (NH_4_)_2_SO_4_ (a-c) and NaCl (d-f).

Run	Space Type	A: pH	B: (NH_4_)_2_SO_4_ concentration (mM)	Binding yield (%)		
(a)						
1	Factorial	4.5	0	41		
2	Factorial	8	0	5		
3	Factorial	4.5	1200	97.5		
4	Axial	6.25	0	12.5		
5	Center	6.25	600	45		
6	Center	6.25	600	37.5		
7	Axial	4.5	600	97		
8	Axial	6.25	1200	87.5		
9	Axial	8	600	35		
10	Factorial	8	1200	80		
11	Center	6.25	600	50		

Source	Sum of Squares	df	Mean Square	F-value	p-value	
(b)						
Model	0.0642	5	0.0128	98.63	< 0.0001	Significant
A-pH	0.0142	1	0.0142	108.79	0.0001	
B-(NH_4_)_2_SO_4_ concentration	0.0408	1	0.0408	313.82	< 0.0001	
AB	0.0063	1	0.0063	48.37	0.0009	
A^2^	0.0007	1	0.0007	5.55	0.0652	
B^2^	0.0027	1	0.0027	20.78	0.0061	
Residual	0.0007	5	0.0001			
Lack of Fit	0.0003	3	0.0001	0.5669	0.6884	Not significant
Pure Error	0.0004	2	0.0002			
Cor Total	0.0648	10				

SD	Mean	C.V. %	R^2^	Adjusted R^2^	Predicted R^2^	Adeq Precision
(c)						
0.0114	1.30	0.8785	0.9900	0.9799	0.9550	31.1161

Run	Space Type	A: pH	B: NaCl concentration (mM)	Binding yield (%)		
(d)						
1	Factorial	4.5	0	41.2		
2	Factorial	8	0	5		
3	Factorial	4.5	2000	93.7		
4	Axial	6.25	0	12.5		
5	Center	6.25	1000	30		
6	Center	6.25	1000	37.5		
7	Axial	4.5	1000	97.5		
8	Axial	6.25	2000	35		
9	Axial	8	1000	12.5		
10	Factorial	8	2000	12.5		
11	Center	6.25	1000	35		

Source	Sum of Squares	df	Mean Square	F-value	p-value	
(e)						
Model	8.22	5	1.64	232.26	< 0.0001	Significant
A-pH	6.36	1	6.36	898.96	< 0.0001	
B-NaCl concentration	1.28	1	1.28	180.43	< 0.0001	
AB	0.0022	1	0.0022	0.3164	0.5980	
A^2^	0.0046	1	0.0046	0.6529	0.4558	
B^2^	0.5585	1	0.5585	78.94	0.0003	
Residual	0.0354	5	0.0071			
Lack of Fit	0.0093	3	0.0031	0.2367	0.8659	Not significant
Pure Error	0.0261	2	0.0131			
Cor total	8.25	10				

SD	Mean	C.V. %	R^2^	Adjusted R^2^	Predicted R^2^	Adeq precision
(f)						
0.0777	3.29	2.36	0.9949	0.9927	0.9893	63.6573

The raw data were analyzed and transformed according to B-C plot recommendation. The raw data for binding rHBsAg on Capto MMC in the presence of (NH_4_)_2_SO_4_ were transformed (power transformation with Lambda value of 0.07) and a full quadratic model (F-value = 98.63) was generated for binding of rHBsAg on Capto MMC resin. The model terms with P-values < 0.1000 were remained. As shown in Table 3b, the Lack of Fit F-value (0.57) is not significant relative to the pure error and the model is therefore fitted to the data correctly. The difference value (less than 0.2) between Predicted R^2^ and Adjusted R^2^ (Table 3c) indicates a reasonable agreement between these values. The Adeq Precision > 4 is desirable and indicates an adequate signal to noise ratio; therefore, this model can be used to navigate the design space (Table 3c).

The raw data for binding rHBsAg on Capto MMC in the presence of NaCl were transformed (Natural Log) and a quadratic model (F-value = 232.26) was generated. The significant and non-significant terms have been demonstrated in Table 3e. Removing the non-significant terms (i.e., A^2^ and AB) from the model generated a reduced quadratic model with better Lack of Fit and improved fit statistics values.

The Lack of Fit F-value of 0.24 was not significant relative to the pure error and the model was then fitted to the data correctly (Table 3e). The Predicted R^2^ of 0.9893 was in reasonable agreement with the Adjusted R^2^ of 0.9927 and the Adeq Precision of 63.657 implied the adequate signal to noise ratio (Table 3f). This model was used to navigate the design space.

**Figure 3:**
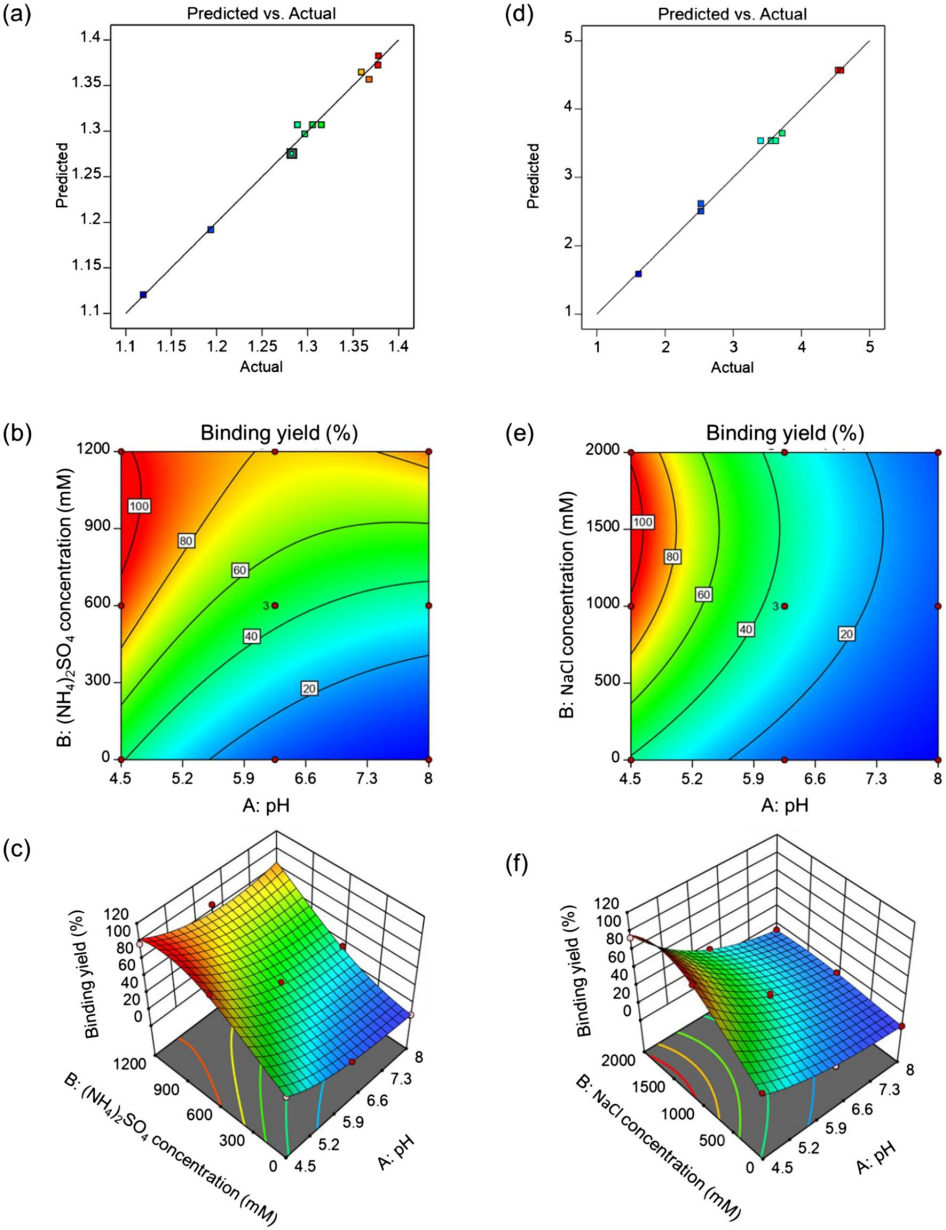
The graphical presentation of rHBsAg binding on Capto MMC resin in the presence of (NH_4_)_2_SO_4_ and NaCl.

Subsequent to the models generation, the predicted versus actual results were evaluated in order to determine how good the generated models predicted the responses in comparison with actual values. According to the results presented in Figs. 3a, and 3d, the distribution of data points uniformly by the 45 degree line shows that the generated models predict the responses properly.

Counter plots and 3D plots demonstrate the response variations as a function of two factors for (NH_4_)_2_SO_4_ and NaCl.

According to the graphical plots for (NH_4_)_2_SO_4_ (Fig. 3b,c), the highest binding yield were obtained in the pH ranges between 4.5 and 5.0, at the salt concentrations above 750 mM. At the same concentrations of (NH_4_)_2_SO_4,_ lower binding yield values were observed with gradual rise in pH value up to 8.0. Based on the counter and 3D plots for NaCl (Fig. 3e,f), the highest binding yields were observed in the pH values between 4.5 and 4.9 and the salt concentrations more than 700 mM. Similar to results for (NH_4_)_2_SO_4_, binding yield was dropped with increase in pH values up to 8.0, at the same concentration of NaCl. rHBsAg binding yield of 100% was attained using both salts at low pH values.

The numerical optimization of Design Expert software (Tables 4a and 4b) suggested that the optimal rHBsAg binding to Capto MMC resin occurred in the pH values near 4.5, at NaCl concentrations above 1000 mM and (NH_4_)_2_SO_4_ concentrations above 800 mM.

**Table 4:** The optimal points for rHBsAg binding on Capto MMC resin in the presence of (NH4)_2_SO_4_ (a) and NaCl (b).

Number	pH	(NH_4_)_2_SO_4_ concentration (mM)	Binding yield (%)	Desirability	
(a)					
1	4.714	1125.564	98.999	1.000	Selected
2	4.613	816.785	99.000	1.000	
3	4.735	1076.725	98.998	1.000	
4	4.705	934.117	99.001	1.000	
5	4.620	824.055	98.999	1.000	
6	4.722	1111.405	98.998	1.000	
7	4.670	1175.054	99.002	1.000	
8	4.738	1054.429	99.003	1.000	
9	4.693	1153.296	98.998	1.000	
10	4.739	1031.973	98.998	1.000	

Number	pH	NaCl concentration (mM)	Binding yield (%)	Desirability	
(b)					
1	4.586	1788.998	98.999	1.000	Selected
2	4.644	1423.234	99.000	1.000	
3	4.545	1138.196	99.001	1.000	
4	4.649	1490.598	98.999	1.000	
5	4.503	1070.220	99.000	1.000	
6	4.517	1915.657	99.000	1.000	
7	4.647	1453.816	99.000	1.000	
8	4.616	1711.452	99.000	1.000	
9	4.649	1535.818	98.999	1.000	
10	4.646	1569.732	99.000	1.000	

The first five optimal points (i.e., the numbers 1 to 5 in Tables 4a and 4b) were chosen for the models’ confirmation.

The robustness of the first optimal point (i.e., pH 4.5 and NaCl concentration of 1800 mM) was evaluated in the pH values ranged between 4.4 and 4.9 and NaCl concentrations of 1600 to 2000 mM. The raw data were analyzed and the half-normal plot indicated no significant terms. Consequently, this study demonstrated the binding design space was robust (supplementary file S1).

### Optimization step for elution of rHBsAg from Capto MMC resin

Based on the unfavorable pH range obtained from the binding optimization studies, pH 8.0 was selected for designing elution experiments. The elution experiments were conducted based on OFAT approach. The eighteen OFAT experiments were evaluated using densitometry analysis of the SDS-PAGE gels. According to the results which are presented in Table 5, elution with 1 M arginine and 3 M KSCN led to about 85–90% rHBsAg recovery from Capto MMC resin.

**Table 5:** The rHBsAg recovery obtained from the OFAT experimental runs.

Run	Treatment	Elution yield (%)
1	Tris pH 8	5
2	Tris pH 8 + 1.5 M KSCN	64
3	Tris pH 8 + 3 M KSCN	85
4	Tris pH 8 + 1 M Arginine	90
5	Tris pH 8 + 2 M Arginine	80
6	Tris pH 8 + 20% ethylene glycol	4
7	Tris pH 8 + 20% dioxane	8
8	Tris pH 8 + 2 M urea	47
9	Tris pH 8 + 0.1% triton	0
10	Tris pH 8 + 1 M Arginine + 20% dioxane	90
11	Tris pH 8 + 1 M Arginine + 1.5 M KSCN	65
12	Tris pH 8 + 1 M Arginine + 20% dioxane + 1.5 M KSCN	63
13	Tris pH 8 + 1 M Arginine + 20% ethylene glycol	50
14	Tris pH 8 + 1 M Arginine + 2 M urea	64
15	Tris pH 8 + 1.5 M KSCN + 2 M urea	48
16	Tris pH 8 + 0.1% triton + 1 M Arginine	74
17	Tris pH 8 + 20% isopropanol	30
18	Tris pH 8 + 1 M Arginine + 20% isopropanol	63

The optimal elution (i.e., run 3 in Table 5) was selected and full factorial design was conducted for the elution robustness study in the pH values ranged between 7.8 and 8.2, at KSCN concentrations of 2.7–3.3 M. The experimental data collected from the execution of the design layout have been presented in supplementary file S2. The experimental data relevant to rHBsAg elution yield and purity were analyzed and the half-normal plot indicated no significant term. As shown in the ANOVA table (supplementary file S2), no model was generated and Lack of Fit was non-significant, meaning that the current data could not generate any model. With regard to two responses including rHBsAg elution yield and purity, the optimized region for the elution of rHBsAg from Capto MMC resin was therefore completely reproducible and robust (supplementary file S2).

### Kinetics of rHBsAg binding on Capto MMC resin

**Figure 4:**
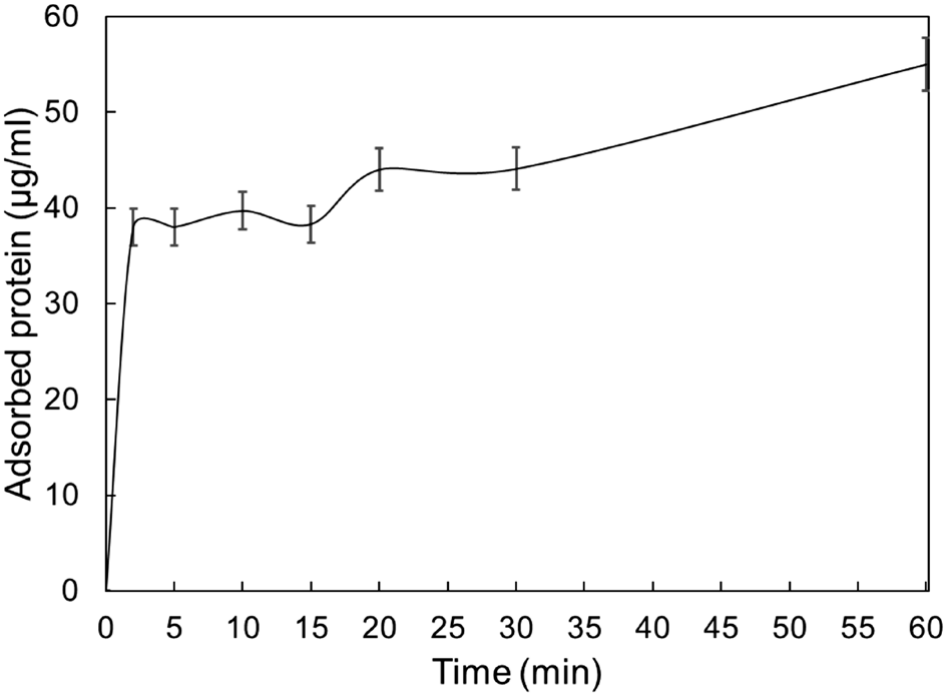
Kinetics of rHBsAg binding on Capto MMC resin.

Recombinant HBsAg binding yield values over various periods of the protein exposure with Capto MMC resin have been demonstrated in Fig. 4. The maximum protein binding (ca. 75%) on the resin occurred in the first 2 min of the exposure.

### Dynamic binding capacity (DBC) of Capto MMC resin for rHBsAg

**Figure 5:**
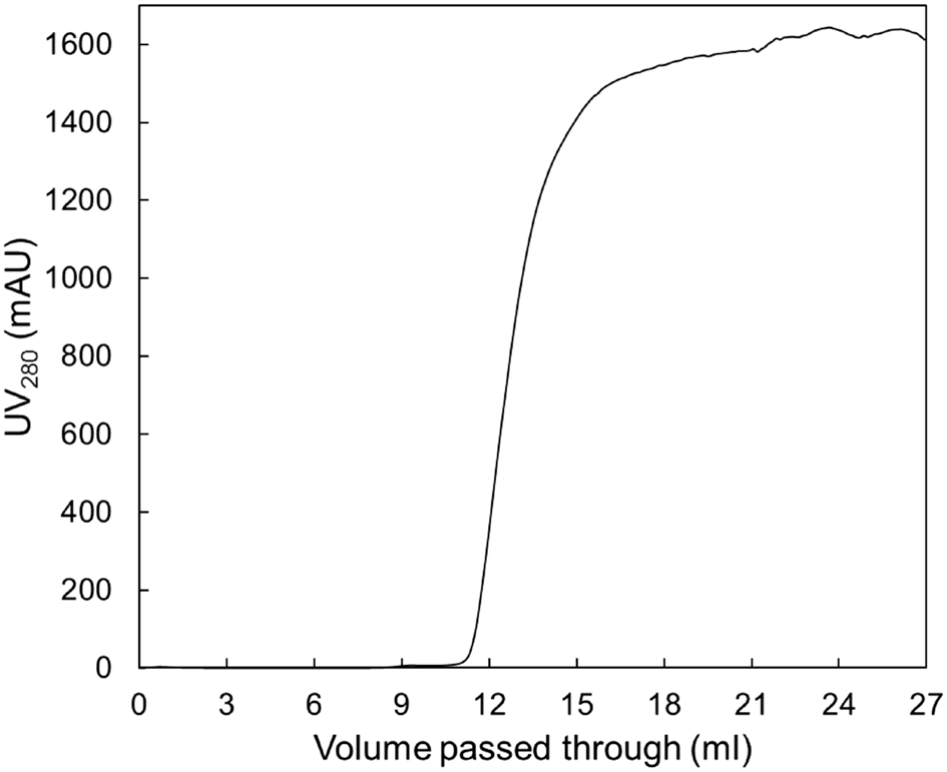
The breakthrough curve of rHBsAg binding on Capto MMC packed column.

DBC is also a very crucial factor for the chromatographic purification process since it affects the number of cycles, the cost and overall productivity of the manufacturing process^30^. In this investigation, DBC of Capto MMC resin was calculated at 10% breakthrough using a packed column (with a bed volume of 5 ml) and an initial target protein concentration of 0.5 mg/ml (Fig. 5).

DBC of some chromatography resins used in the purification of rHBsAg has been reported^31^. By comparing these resins, DBC_10%_ of Capto MMC (0.43 mg/ml) is close to the binding capacity of IAF (0.47 mg/ml) and much higher than that of HIC (0.085 mg/ml)^31^.

### Purification of rHBsAg using Capto MMC packed column

**Figure 6:**
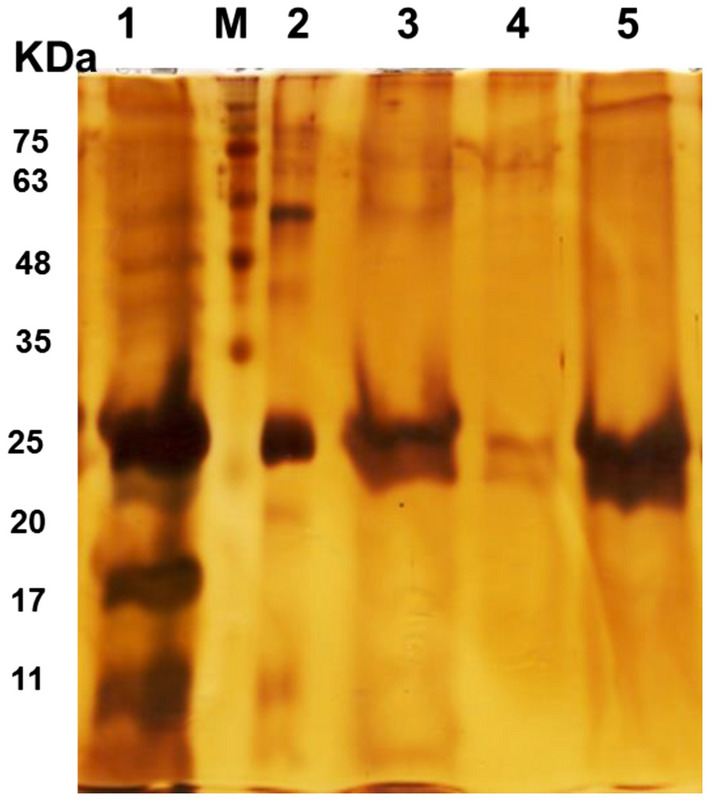
SDS-PAGE analysis of various fractions collected throughout rHBsAg purification using Capto MMC packed column from the feedstock prepared by dialysis in 20 mM sodium acetate, 1800 mM NaCl, pH 4.5. Lanes: M—Molecular weight markers; 1—Load; 2—cAPI, 3—Elution, 4—Wash, 5—Flow-through.

During rHBsAg purification by Capto MMC packed column, 20 ml of feedstock sample (dialyzed in 20 mM sodium acetate, 1800 mM NaCl, pH 4.5) with a rHBsAg content of 3.28 mg and a total protein content of 9.54 mg was loaded on the column. Under such condition, rHBsAg with 100% purity was obtained in the flow-through and elution fractions (Fig. 6).

### Purification of rHBsAg using unfavorable conditions for binding of rHBsAg on Capto MMC resin

**Figure 7:**
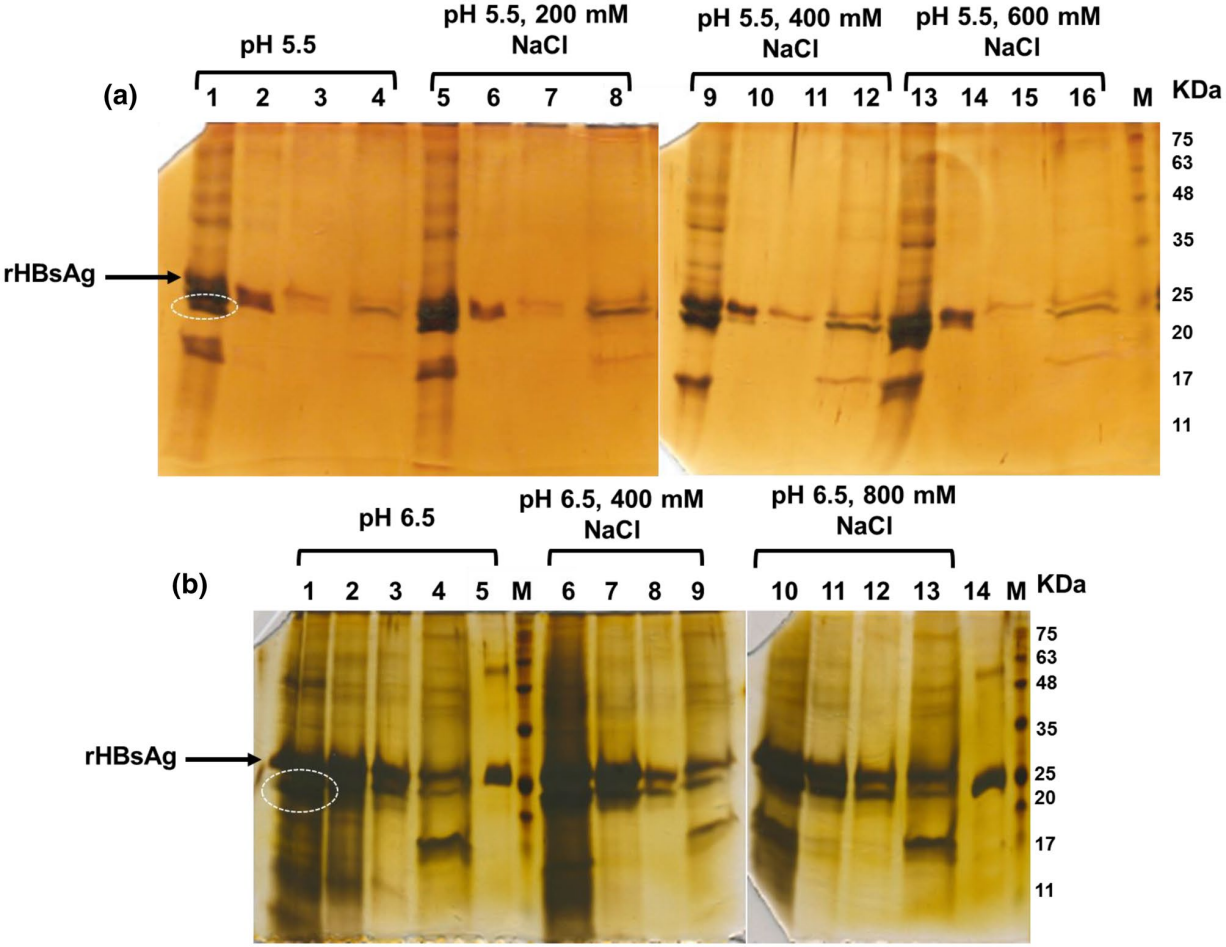
SDS-PAGE analysis of various fractions collected throughout rHBsAg purification using Capto MMC resin in flow-through mode. (**a**) Purification done at pH 5.5, Lanes: M—Molecular weight markers; 1, 5, 9, 13—Load; 2, 6, 10, 14—Flow-through; 3, 7, 11, 15—Wash; 4, 8, 12, 16– Elution fraction. (**b**) Purification done at pH 6.5, Lanes: M– Molecular weight markers; 5 and 14—cAPI; 1, 6, 10—Load; 2, 7, 11—Flow-through; 3, 8, 12—Wash; 4, 9, 13—Elution fractions.

These experiments were conducted consecutively. The preliminary experiments (supplementary file S3) were carried out to determine optimal conditions for binding “protein impurities” (instead of rHBsAg) to the resin (at various NaCl concentrations ranged between 400 and 1600 mM and pH 5.5). Then based on the favorable conditions achieved from this set of experiments for binding “protein impurities” to the resin, further experiments were done (at and around the selected conditions). The later experiments for flow-through mode purification of rHBsAg were performed using samples buffered at pH 5.5 and 6.5. In the work done at pH 5.5, almost all protein impurities in the sample bind tightly to the Capto MMC resin in NaCl concentrations ranging from 0 to 600 mM, and rHBsAg was mostly appeared in the flow-through fraction with high purity (> 80%) (Fig. 7a). In the work done at pH 6.5, binding of protein impurities (particularly those with molecular weights lower than rHBsAg) to Capto MMC resin was substantially increased with rise in NaCl concentration. With an increase in NaCl concentration from 0 to 800 mM, the purity of rHBsAg obtained in the flow-through fraction was also augmented from 50% to above 73% (Fig. 7b).

**Figure 8:**
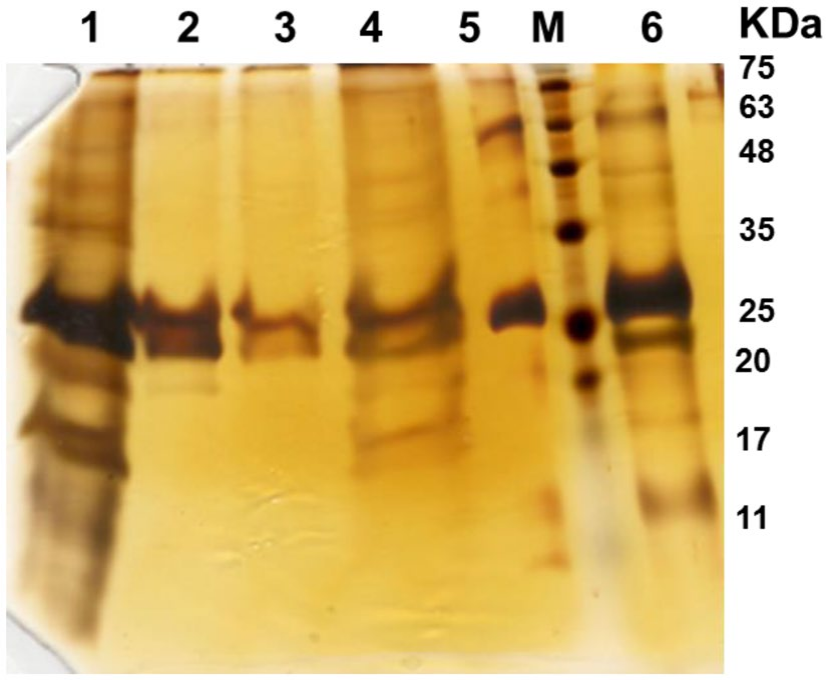
SDS-PAGE analysis of various fractions collected throughout rHBsAg purification using Capto MMC resin in flow-through mode at pH 5.5, 600 mM NaCl. Lanes: M—Molecular weight markers; 1—Load; 2—Flow-through; 3—Wash; 4—Elution fractions; 5—cAPI; 6—Concentrated flow-through.

Following conducting these experiments, final experiment on flow-through purification of rHBsAg was done with selecting pH 5.5 and 600 mM NaCl as optimum conditions, and the purified protein was concentrated using 100 kDa Amicon-centrifugal filter unit. As illustrated in Fig. 8, rHBsAg obtained after concentration had a purity of 90 ± 5% and a recovery of 84 ± 1%.

### Characterization of rHBsAg purified by Capto MMC resin

The final product quality is the foremost parameter in process optimization studies using QbD^32^. Hence, for the purpose of IAF (or IAF plus other chromatographic purification steps) replacement with Capto MMC packed column, the same rHBsAg quality is required. To obtain sufficient protein for such quality characterizations, rHBsAg was purified in bind-elute mode (with almost 100% purity) from the feedstock using Capto MMC packed column.

### Purity and particle size distribution analyses

**Figure 9:**
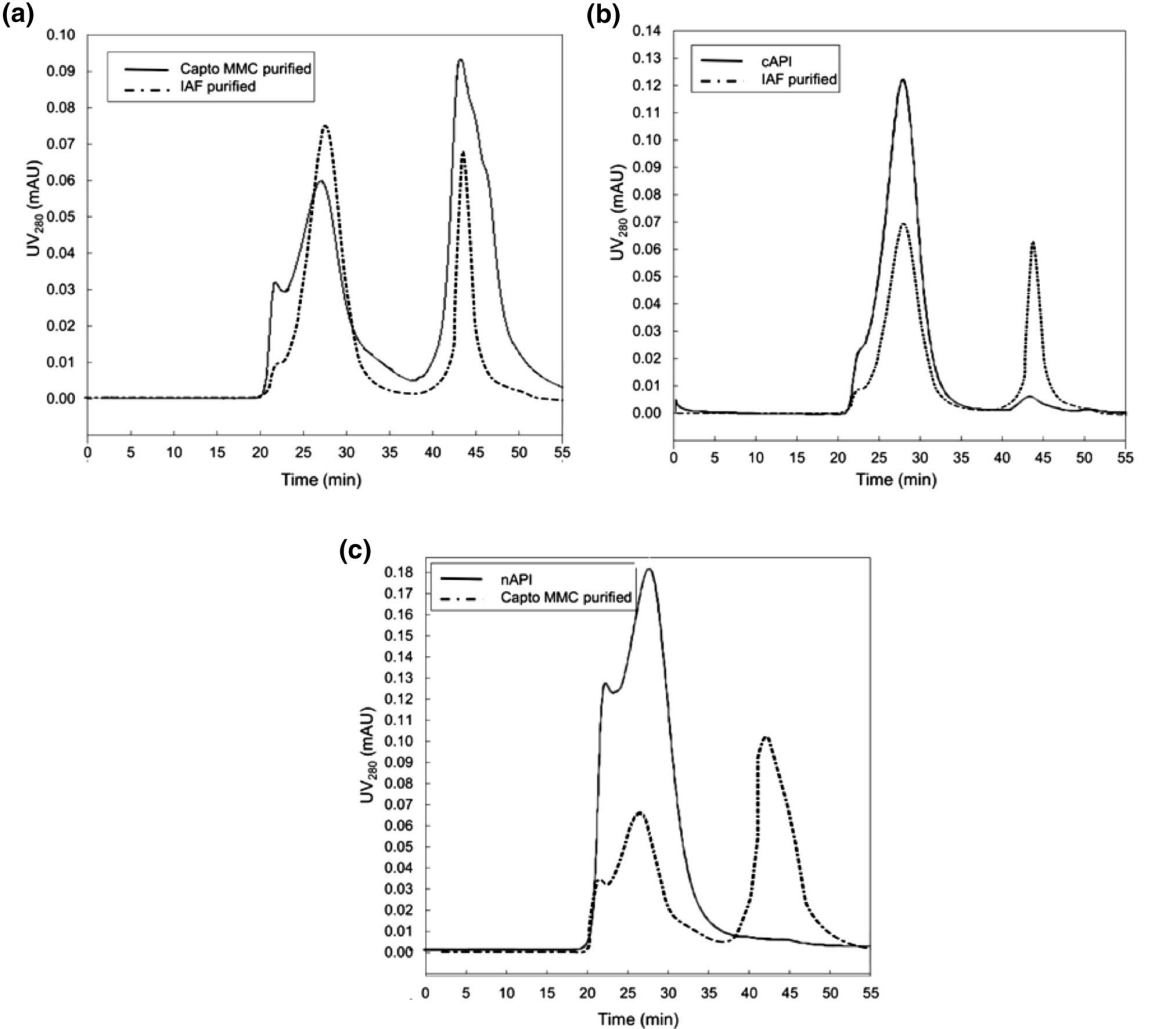
SEC-HPLC analysis for particle size distribution of rHBsAg in various samples. (**a**) Capto MMC and immunoaffinity purified rHBsAg, (**b**) Immunoaffinity eluate and cAPI sample derived from the stabilization, gel filtration, Ion exchange chromatography and ultrafiltration, (c) Capto MMC purified and nAPI derived from the stabilization and ultrafiltration. Column: TSKgel 5000 PW with bed dimensions of 7.5 mm diameter × 60 cm. Injection: 200 μl. Mobile phase: PBS (1X, pH 7.4). Flow rate: 0.5 ml/min. Temperature: 25 °C.

As declared in Table 2, purity and particle size distribution are two CQAs in IAF step during large-scale rHBsAg manufacturing. In order to investigate these CQAs, the Capto MMC purified rHBsAg and the elution sample obtained from the large scale IAF were analyzed using SEC-HPLC and compared (Fig. 9a). According to these results, the Capto MMC peaks with retention times identical to the IAF eluate (i.e., 22, 27 and 43 min) were observed. The comparison of SEC-HPLC profiles of these samples also confirmed the high purity measured by SDS-PAGE for the Capto MMC purified fractions (99 ± 1). The host cell residual DNA in the Capto MMC purified sample was also quantified by qPCR and its concentration was about 3 ± 0.5 pg/20 µg rHBsAg.

In the conventional downstream processing for manufacturing of rHBsAg-API, the purified rHBsAg by IAF is stabilized in the presence of Tris HCl, pH 7.2 (containing 3 M KSCN, 2 M NaCl, 3 mM EDTA, at 60 °C) and next passed through sequential gel filtration-ion exchange chromatography (for elimination of the immunoaffinity resin-leaked IgG antibodies and residual DNA impurities) and ultrafiltration^29^.The results of SEC-HPLC analysis of the IAF eluate and cAPI have been shown in Fig. 9b. As seen in this figure, the retention time of the main peak of cAPI sample is 27 min. Previously, the similar results on SEC-HPLC analysis of cAPI have been also reported^33,34^. Due to the absence of mouse IgG and insignificant residual DNA in the Capto MMC purified sample, sequential gel filtration-ion exchange chromatography (being used in the conventional process of cAPI preparartion) was disregarded in the process of nAPI preparation, and only the stabilization plus ultrafiltration steps (mentioned in Fig. 1 and supplementary file S4) were utilized. SEC-HPLC profile of nAPI has been demonstrated in Fig. 9c and supplementary file S5. As it is evident from Fig. 9b, c, the protein particle size distribution in nAPI (88.83 ± 2.49) is largely identical to cAPI.

Enzyme immunoassay was utilized to determine the exact concentration of the yeast HCP in nAPI and cAPI. The HCP concentration in nAPI was estimated about 39.1 ± 1.9 ng/mg rHBsAg, which was lower than the value related to cAPI (56.3 ng/mg rHBsAg).

### Circular dichorism, specific activity and in vitro relative potency assay

CD is an appropriate method for analysis of the secondary structures of proteins in solution in the far UV-region (between 240 and 200 nm)^35,36^. In the present work, CD was employed to study the secondary structures of cAPI and nAPI. The samples were dialyzed against sodium phosphate buffer in order to eliminate optically active substances which absorb strongly in the far-UV range. Various secondary structures in proteins generate distinctive CD spectra in the in the far-UV region^37,38^.

**Figure 10:**
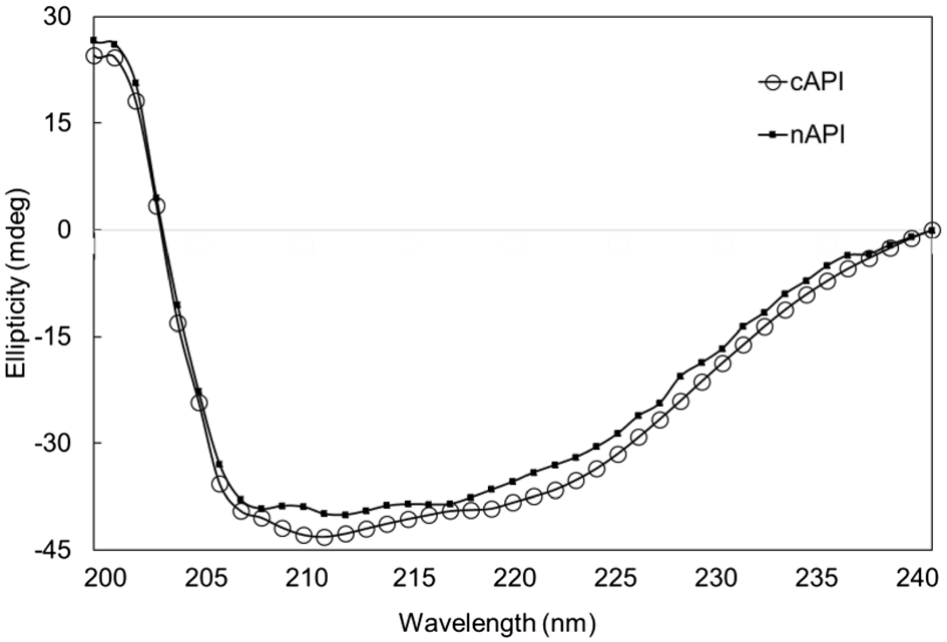
Far-UV CD spectra of cAPI and nAPI (0.15 mg/ml in 15 mM sodium phosphate buffer, pH 7.0). The spectra were recorded in 0.1 cm path length cell using Jasco J-810 spectropolarimeter at a scan speed of 200 nm/min and a bandwidth of 1.5 nm.

The CD spectra of cAPI and nAPI have been illustrated in Fig. 10, and it is evident that the spectra peaks have similar patterns in the far-UV region. The content of secondary structure elements of cAPI and nAPI have been mentioned in Table 6.

**Table 6:** The percentage of rHBsAg secondary structures in cAPI and nAPI.

Fraction of secondary structure (%)	cAPI	nAPI
α-Helix	29.3%	32.9%
β-sheet	14.4%	4.9%
Turn	28.0%	36.1%
Random	28.3%	26.0%
β-sheet + Turn + Random	70.7	67.1%

**Figure 11:**
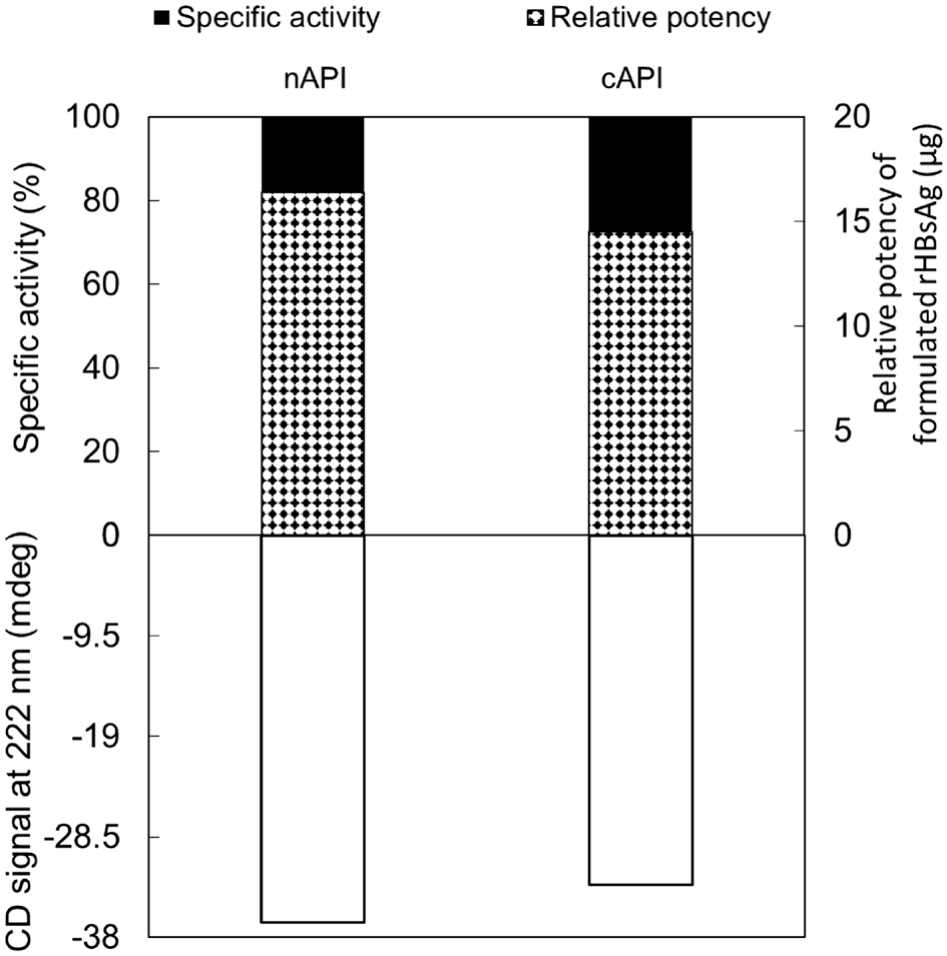
Specific activity and relative potency values in correlation with CD intensity at 222 nm for cAPI and nAPI samples.

The activity and in vitro relative potency of cAPI and nAPI were further quantified and compared. Like cAPI, the specific activity of nAPI was 100% following antibody sandwich enzyme immunoassay. Subsequent to the formulation of cAPI and nAPI in aluminum hydroxide, the relative potency of the samples was quantified using antigen sandwich enzyme immunoassay. Figure 11 demonstrates the specific activity and relative potency values in correlation with CD intensity at 222 nm which is thought to reflect the presence of α-helix in the samples. As it is evident from this figure, the relative potency of nAPI is about 12% more than that of cAPI, which can be related to a greater CD intensity at 222 nm in nAPI. These results are in agreement with those reported by Vanille J. Greiner and et al.^39^.

### Cleaning and reutilization of Capto MMC resin

**Figure 12:**
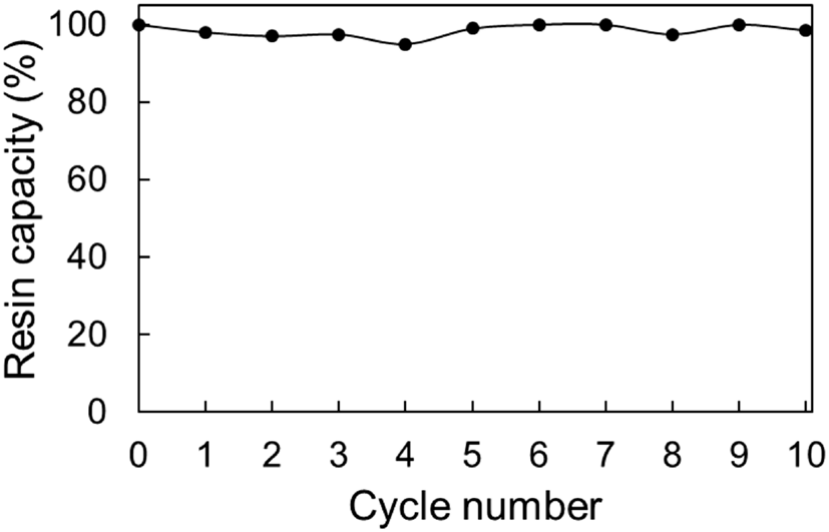
Capto MMC capacity for rHBsAg in ten successive adsorption–elution–cleaning cycles. The value of 0.55 mg/ml which was obtained in the first binding experiment was considered a 100% capacity.

Following target protein adsorption–elution processes, the chemical cleaning of the fouling agents (i.e., product or host cell–related contaminants) from the resin without damaging its binding capacity is a very important factor for the success of the chromatographic purification method development. In this investigation, 1 M NaOH, 0.1% Tween 20 and 2 M NaCl were used to remove host cell–related contaminants from Capto MMC resin subsequent to the elution step. Repeated use of Capto MMC resin in ten adsorption–elution–cleaning cycles indicated that 97–100% of the resin capacity was maintained (Fig. 12).

## Discussion

As mentioned in the results presented in Fig. 2 and Table1, rHBsAg purity achieved by buffer exchanging in sodium acetate, pH 4.5, was 2.7 times higher than that obtained in Tris–HCl buffer at the same pH value. Acetate is an anion which is located in the left side of the Hofmeister series; thereby, it is able to induce “salting out” and precipitation effects. Whereas NO_3_ of Tris buffer located in the right side of the Hofmeister series, which can lead to “salting in” effects^19^. Previously, sodium acetate buffer has been used in high concentrations and under specific pH conditions to precipitate protein impurities^40^. Overall, with providing a specific combination of different conditions such as KSCN concentration reduction, pH and buffering adjustments for the SAP sample, a high level of impurities precipitation and separation (up to 73%) can be achieved.

To further define design space for rHBsAg purification using Capto MMC resin, CQAs including purity, particle size distribution, specific activity, potency, residual HCD and HCP were selected to be assessed in this investigation. Aggregation has substantial effects on biological efficacy, immunogenicity, safety and activity of rHBsAg, and they are considered as one of the major origins of the product instability^41–44^. It has been also demonstrated that 22 nm VLPs of rHBsAg, being assembled from the protein monomers throughout maturation in downstream operations, are highly immunogenic due to achieving native like conformation^45^; therefore, greater immunogenicity is reached via higher rate of rHBsAg VLPs formation. Moreover, host-related impurities such as HCD and HCP residues are capable to provoke immunogenicity or modify the product functionality^42,46,47^.

In the optimization step for rHBsAg binding on Capto MMC resin, a protein binding yield of 100% was achieved in the design of experiments using (NH_4_)_2_SO_4_ (Table 4a) and NaCl (Table 4b). NaCl has been used in the conventional large-scale chromatographic steps (i.e., 1^st^ anion exchange and immunoaffinity chromatography shown in Fig. 1) in the downstream processing of rHBsAg; hence, this salt was used for rHBsAg binding on Capto MMC in further experiments (to avoid additional buffer exchange for adjusting binding condition).

In this study, the area of variation between upper and lower limits of the DOE factors were considered as wide as possible in order to avoid optimal points missing and get insightful understanding around the process design space. For instance, based on the results of binding experiments (Fig. 3), the optimal binding space was defined and also it was revealed that pH had very dominant effect on rHBsAg binding pattern of Capto MMC resin.

The effect of pH on adsorption of several proteins by Capto MMC resin has been previously investigated and it has been also demonstrated that the proteins adsorption substantially dropped with rise in pH towards 8.0^48^. Given that the PI of rHBsAg is about 4.2^33^ and based on the cation-exchange properties of Capto MMC resin, it is expected that the alterations in the protein net charge and hydrophobicity with increase in pH beyond PI lead to reduced adsorption to the ligand. The unfavorable pH value around 8.0 was further used in designing elution experiments.

The results of elution experiments, showed that arginine was the best elution agent for releasing rHBsAg from Capto MMC resin and this was in consistent with other studies conducted for eluting various proteins from this resin^49^. The elution performance of arginine corresponds to its multimodal interactions with Capto MMC resin (i.e., electrostatic interaction, hydrophobic interaction or hydrogen bonding)^49^. However, 3 M KSCN was chosen for further investigations due to the fact that it has been used in elution of rHBsAg from immunoaffinity resin and essentially incorporated for rHBsAg stabilization in the conventional large-scale downstream operation^45,50^. In fact, denaturing effect of the 3 M KSCN results in formation and rearrangement of rHBsAg intermediate isomers which are more stable under these denaturing and following native conditions^45,50^.

The purity of the recovered rHBsAg was increased from 36 to 100% with a purification fold of 2.77. The high purity of the recovered proteins from multimodal resins originates from their high selectivity^26^. The capability of multimodal resins in reducing impurities has been shown in several studies^51–53^. For example, a target protein purity more than 97.5% was achieved in an investigation using sequential precipitation-Capto MMC purification ^52^.

Results of rHBsAg purification using Capto MMC packed column show that rHBsAg with nearly 100% purity can be obtained in both flow-through and elution fractions. The lack of impurities in the flow-through and elution fractions can be corresponded to their stronger interactions with the resin, compared to rHBsAg, so that they are not released from the column with the applied elution condition. The recovery of rHBsAg (with 100% purity) in the flow-through and elution fractions was 66% and 33%, respectively. This demonstrates the high selectivity of multimodal Capto MMC resin which discriminates between rHBsAg and protein impurities in the applied elution conditions.

In order to reveal interactions of rHBsAg and protein impurities in the feedstock sample with the resin under other buffering conditions, further experiments were conducted at pH values above 4.5 and NaCl concentration lower than 800 mM (i.e., the conditions unfavorable for binding of rHBsAg on Capto MMC resin (Fig. 3e).

According to the results presented in Fig. 7, the recovery of rHBsAg in the flow-through fraction at pH 6.5 was slightly greater than that achieved at pH 5.5, but the purity of obtained target protein was higher at pH 5.5. The main impurity which remains in the flow-through fraction at pH 6.5 is the one with close molecular weight to rHBsAg (circled in Fig. 7). By rising pH from 5.5 to 6.5, the strength of interactions between protein impurities and Capto MMC resin was reduced. It seems that interactions for binding protein impurities to Capto MMC multimodal resin are hydrophobic, as hydrophobic interactions are more induced by pH reduction and salt concentration enhancement.

Referring to the results of protein impurities removal (Fig. 2), buffer exchange of the SAP in 20 mM sodium acetate, pH 4.5, can eliminate major bulk impurities including the one circled in Fig. 7. Hence, it can be construed that through the elimination of the circled impurity by the buffer exchange at pH 4.5 and subsequent purification using Capto MMC at higher pH (e.g., 5.5), further increase in the purity of obtained rHBsAg would be possible.

The whole results for purification of rHBsAg from feedstocks prepared in different buffering conditions (i.e., buffer exchange step) using various amounts of Capto MMC resin in different modes have been reported in Table 7.

**Table 7:** Results obtained from sequential buffer exchange and various modes of Capto MMC purification.

Process	Recovery (%)* obtained from Capto MMC purification in the mode: BE^a^ FT^b^	rHBsAg purity (%)	Purification fold (PF) obtained from Capto MMC purification**	Capto MMC binding capacity (mg/ml) for: rHBsAg HCPs^c^	Resin/Feedstock ratio	Total process recovery (%) ***	Amount of rHBsAg (mg) purified in the process using 1 ml resin
Buffer exchange and BE purification mode, both conducted in sodium acetate, pH 4.5	82.5 ± 0.7 NA	99 ± 1	2.77	0.17 0.314	1	65.17	0.140
Buffer exchange and BE purification mode, both conducted in sodium acetate, pH 4.5	29.37 ± 2 66 ± 0.5	99 ± 1	2.77	0.22 ≥ 1.25	0.25	75.34	0.642 (0.440 in FT, 0.202 in elution
Buffer exchange in sodium acetate, pH 4.5, and FT purification mode in sodium acetate, pH 5.5	NA 84 ± 1	98 ± 2	2.77	0.04 ≥ 1.25	0.25	66.36	0.554
Buffer exchange and FT purification mode, both conducted in sodium acetate, pH 5.5	NA 84 ± 1	90 ± 5	6.76	0. 04 ≥ 1.3	1	72.24	0.153
Buffer exchange in sodium acetate, pH 5.5, and FT purification mode in sodium acetate, pH 6.5	NA 88 ± 1	77 ± 1	5.78	0.02 ≥ 1	1	75.68	0.161

^a^Flow-through.

^b^Bind-elute.

^c^Host cell proteins.

*Calculated by dividing amount of rHBsAg in the respective fraction by amount of rHBsAg in the feedstock.

**Calculated by dividing rHBsAg purity after Capto MMC purification step by rHBsAg purity in the feedstock.

***Calculated by multiplying rHBsAg recovery achieved following buffer exchange by rHBsAg recovery obtained after Capto MMC purification.

*NA* Not applicable.

According to the results presented in Table 7, due to elimination of most impurities via buffer exchange of the SAP in 20 mM sodium acetate, pH 4.5, it is possible to obtain much more rHBsAg (up to 4.3-fold) with 100% purity and using much less resin. In comparison, the capacity of Capto MMC resin for rHBsAg purification from feedstock is considerably limited by much higher protein impurities remained following buffer exchange of the SAP in 20 mM sodium acetate, pH 5.5.

Owing to the absence of mouse IgG and insignificant residual DNA in the Capto MMC purified sample, there is no need to perform extra chromatographic steps to reach active pharmaceutical ingredient (API). Hence the purified rHBsAg was used for further characterization studies.

In the CD spectroscopy analysis, the α-helix content of nAPI was 3.5% higher than cAPI. It has been reported that increase in rHBsAg α-helix content leads to rise in antigenicity and immunogenicity ^39^. On the other hand, β-sheets, turn and random structures are responsible for protein aggregation^36,41^. The sum of these structures in cAPI is greater than that of nAPI (Table 6); hence, nAPI may be less prone to aggregation. Therefore, from these structural aspects, it can be concluded that nAPI with slightly modified secondary structure may show superior quality attributes in terms of aggregation, biological activity, antigenicity and immunogenicity. The relative potency of nAPI was about 12% more than that of cAPI, which confirmed the relation between α-helix content and rHBsAg antigenicity. Similar results have been also previously reported by Vanille J. Greiner et al.^39^.

## Conclusion

In the conventional production process of rHBsAg, a VLP-base protein used for manufacturing recombinant hepatitis B vaccine, a huge amount of protein impurities (90%) remains in the feedstock following acid precipitation step (namely semi-purification); consequently, a feed stream with low purity of HBsAg (of about 5%) is resulted in. Also, the large size of the VLPs leads to low chromatographic resin performance with respect to capacity and recovery. Collectively, these issues make the downstream processing of the VLPs very challenging and costly. Throughout preliminarily experiments conducted in the current study, a high level reduction in protein impurities (up to 73%) was achieved by selecting specific buffering conditions (e.g., 20 mM sodium acetate, pH 4.5) and concomitant removal of KSCN from the sample. This reduction in protein impurities can substantially increase the efficiency of the subsequent chromatographic purification method in both bind-elute and flow-through mode. Following appropriate process optimization using DOE (RSM) and OFAT experiments, the conditions for binding and release of rHBsAg on Capto MMC resin were determined and such conditions were applied for designing of rHBsAg purification process in both bind-elute and flow-through modes. Consequently, rHBsAg with 100% purity and 65–100% recovery was obtained. Under optimized protein impurities reduction and chromatographic process conditions, it was revealed that capability of Capto MMC resin for purification of rHBsAg in the flow-through mode was 3.5 times higher than the bind-elute mode. With regard to purity, recovery and process capability, the quantities reported in this study were far greater than those reported elsewhere for immunoaffinity in the conventional downstream processing^31^. It was also disclosed that the quality attributes (particle size distribution, HCD, HCP, secondary structures, specific activity and relative potency) of the rHBsAg, purified based on the achieved optimized process in this study, were similar or superior to those of the standard samples (i.e., samples derived from the conventional industrial process).

Overall, the newly developed DSP of rHBsAg in this study (which includes new semi-purification step for efficient reduction of protein impurities and Capto MMC chromatographic purification step) can be considered as a good replacement for the conventional DSP of rHBsAg, due to advantages such as superior target protein quality, long-lasting resin efficacy, shorter and less expensive downstream process (supplementary file S4). Moreover, the newly developed DSP of rHBsAg has the potential to replace the burdensome immunoaffinity resin with Capto MMC resin. This process would be also employable for purification of both non-VLP—and VLP—based target proteins expressed in the yeast.

## Methods

### Sample preparation

*P. pastoris* crude extract containing rHBsAg (i.e., mechanically disrupted yeast cells in 20 mM Tris–HCl, 3 M KSCN, pH 7.2) and conventional rHBsAg API (cAPI) were obtained from Pasteur Institute of Iran. Then 1 M HCl was added to the crude extract until the pH was adjusted on 4.5. Following centrifugation at 10,000* g* for 15 min, the precipitate was separated by centrifugation. The supernatant of acid precipitation (SAP) was used in further studies.

### Investigation of the effect of KSCN removal on the reduction of protein impurities in different buffering conditions

SAP was dialyzed overnight at different pH values ranged between 4.5 and 6.5 using buffers including sodium acetate, Tris–HCl and sodium phosphate. Subsequent to dialysis, the samples were centrifuged at 10,000 *g*, for 15 min and the resulting pellets and supernatants were analyzed using SDS–polyacrylamide gel electrophoresis (SDS-PAGE) and Lowery assay to measure rHBsAg purity and recovery.

### Defining CQAs, CPPs, and CMAs

To deconvolute process complexities in bind-elute as well as flow-through modes, the CQAs and other critical parameters such as CPPs and CMAs were characterized in the design space definition step.

Based on the established product quality control in the manufacturing system, the CQAs related to the DSP of rHBsAg were determined. As the intention of this research preliminary was to replace the IAF chromatography step in rHBsAg downstream processing, the CQAs related to this step were remarked. In order to characterize design space, salt type/concentration and pH were considered as CMAs and CPP, respectively. The parameters including temperature and feedstock concentration were kept constant throughout DOE experiments.

### Optimization step for rHBsAg binding on Capto MMC resin

Subsequent to defining design space elements, binding experiments were performed based on DOE method and using rHBsAg-API. The DOE experiments in the optimization studies were conducted in the pH values ranged between 4.5 and 8.0, NaCl concentrations of 0 to 2000 mM and (NH_4_)_2_SO_4_ concentrations of 0 to 1200 mM.

The design layouts were created using Design-Expert version 11 (StatEase). The central composite face-centered (CCF) design with 2 factors of pH and salt concentration in 3 levels were separately created for NaCl and (NH_4_)_2_SO_4_ (22 runs were outlined). The amount of adsorbed protein (i.e., binding yield percentage) was considered as the DOE response in these DOE designs. To carry out the designed experiments according to the experimental layouts, protein samples were prepared in the respective conditions (in terms of pH and salt type/concentration) and Capto MMC resin was aliquoted in 22 Eppendorf microtubes in equal volumes (200 µl). Each microtube containing Capto MMC resin was equilibrated with the relevant binding buffer. The protein samples (200 µl) were then added to the appropriate equilibrated resin in the Eppendorf microtube. The microtubes were left at room temperature for 1 h, with mixing at 5 min intervals. The microtubes were finally centrifuged at 300*g* for 2 min and the protein solutions (supernatants) were separated for further SDS-PAGE and densitometry (using Image Lab Software). The amount of adsorbed rHBsAg in each condition was calculated by subtracting initial protein concentration from final protein concentration. The obtained data were transferred to the DOE software, the suitable transformation was selected and the most fitted model for the experimental data was generated. Subsequent to the model generation, the optimal point was defined by numerical optimization and verified experimentally. To ensure about the reproducibility and robustness of the binding yield obtained through the protein binding optimization, robustness study around the selected optimal binding point (achieved from the numerical optimization) was carried out using full factorial design (FFD).

### Optimization step for elution of rHBsAg from Capto MMC

For this purpose, eighteen OFAT experiments were designed using single and combined chemicals (including KSCN, arginine, ethylene glycol, dioxane, urea, Triton X-100 and isopropanol made in 20 mM Tris, pH 8.0) at different concentrations. To perform these experiments, a protein sample (1800 µl) was prepared in the optimum binding point condition (acquired from the optimization study done earlier) and added to 1800 µl Capto MMC resin. Subsequent to the incubation of the mixture for 1 h at room temperature, the supernatant was separated from the resin by centrifugation at 300 *g* for 2 min. The resin was washed with the equilibration buffer and aliquoted in 18 Eppendorf microtubes in equal volumes (100 µl). For rHBsAg elution based on the OFAT design, 300 µl of each treatment was added to the resin in each microtube for 15 min with continuous mixing. The resin in each microtube was separated from the solution using centrifugation at 300*g* for 2 min and the supernatants were used for further SDS-PAGE and densitometry analyses. The amount of eluted rHBsAg (i.e., elution yield percentage) and its purity were considered as the responses in the elution optimization experiments. To ensure about the reproducibility and robustness of the elution yield obtained through the protein elution optimization, robustness study around the selected optimal elution point (Tris–HCl, pH 8.0 + 3 M KSCN) was carried out using full factorial design (FFD).

### Kinetics of rHBsAg binding on Capto MMC resin

For this purpose, cAPI (1400 µl) was prepared in the optimum binding point condition achieved from the binding numerical optimization. The Capto MMC resin was washed three times with the equilibration buffer, aliquoted into 14 Eppendorf microtubes in equal volumes (90 µl). The resin was separated from the equilibration buffer in each microtube, by centrifugation at 300*g* for 2 min, and 100 µl of the protein sample was added to each microtube. The microtubes were then left at room temperature for various periods (2, 5, 10, 15, 20, 30 and 60 min) with continuous mixing. The experiments were executed in duplicates. Subsequent to the incubations, the supernatants were quickly separated from the resins by spinning for 15 s and used for further SDS-PAGE and densitometry analyses. The protein binding yield percentage in each condition was calculated by subtracting initial protein concentration from final protein concentration.

### Dynamic binding capacity (DBC) of Capto MMC for rHBsAg

A glass column with adjustable plunger was packed with 5 ml Capto MMC resin at a flow rate of 5 ml/min. The column was equilibrated with 20 mM sodium acetate, 1800 mM NaCl, pH 4.5. A protein sample with concentration of 0.5 mg/ml, prepared in the equilibration buffer using pure rHBsAg, was loaded on the column at a flow rate of 0.3 ml/min. The sample loading was continued until the UV_280_ signal reached the plateau. The resin dynamic binding capacity (in 10% breakthrough) for rHBsAg was calculated as below^54^:

$$DBC_{10\% } = \, C_{0} \left( {V_{break10\% } - \, V_{delay} } \right)/BV$$

where DBC_10%_ is the dynamic binding capacity (mg/ml) in 10% breakthrough volume, C_0_ is the initial concentration of protein in solution (mg/ml), V_break10%_ is the volume in 10% breakthrough, V_delay_ is the system delay volume (ml), and BV is the bed volume (ml).

### Purification of rHBsAg in flow-through mode

Based on the results obtained from the rHBsAg binding optimization studies, the unfavorable pH ranges for the protein binding (i.e., nonbinding conditions) were determined. Further experiments were conducted using feedstocks prepared at pH 5.5 and 6.5 with NaCl concentrations ranged between 0 and 800 mM. During these experiments, 300 µl of Capto MMC resin was mixed with 300 µl of the feedstock samples in defined conditions for 1 h. After incubation, the supernatants were separated from the resin by centrifugation at 300*g* for 2 min. The resin in each microtube was washed with 300 μl of the equilibration buffer and then with 300 μl of Tris–HCl, 3 M KSCN, pH 8.0. The resin in each microtube was separated from the supernatant by centrifugation at 300*g* for 2 min, and the supernatants were used for SDS-PAGE and densitometry analyses.

### Purification of rHBsAg from the feedstock by Capto MMC packed column

The glass column packed with 5 ml Capto MMC resin was equilibrated with 20 mM sodium acetate, 1800 mM NaCl, pH 4.5. Twenty-seven milliliters of the feedstock, prepared in the equilibration buffer, was loaded on the column at a flow rate of 0.3 ml/min. The column was then washed with 20 mM acetate buffer (pH 4.5) and elution was made using 20 mM Tris–HCl, 3 M KSCN, pH 8.0. The purified rHBsAg was used for further characterization studies.

### Heat stabilization and concentration of rHBsAg originated from Capto MMC resin (nAPI preparation)

The purified rHBsAg was prepared (by dialysis) in Tris HCl, pH 7.2, containing 3 M KSCN, 2 M NaCl and 3 mM EDTA to fulfill the stabilization process in accordance with large-scale rHBsAg manufacturing. The protein solution was heated to 60 ºC for 2 h. The sample was dialyzed against 15 mM phosphate buffer, pH 6.7, and concentrated using a 100 kDa Amicon-centrifugal filter unit (UFC510008, Merck Milipore, Burlington, Massachusetts, United States) to reach a concentration of 1 mg rHBsAg API/ml.

### Cleaning and reutilization of Capto MMC resin

Following each sorption/elution cycle, cleaning of the product-related contaminants from Capto MMC column was done based on the resin chemical stability specifications provided by the supplier (GE Healthcare, Chicago, Illinois, United States). Briefly, the column was sequentially washed with 1 M NaOH for 1 h, double distilled water (DDW) until NaOH removal, 0.1% Tween 20 for 1 h, DDW until Tween 20 removal, 2 M NaCl for 15 min, 20 mM Tris–HCl (pH 10), and finally DDW. Subsequent to the cleaning fulfillment, the resin (100 µl) was re-equilibrated and mixed with 60 µg of API under optimal binding conditions. After 1 h exposure, the resin was separated from the solution by centrifugation at 300*g*, for 2 min. The samples were analyzed using SDS-PAGE. The cleaning-binding experiments were performed in ten cycles.

## Analytical methods

### SDS–polyacrylamide gel electrophoresis (SDS-PAGE)

SDS-PAGE was performed on 12% gels at 85 V for 3.5 h using Tris–glycine running buffer. Prior to the electrophoresis, all samples were diluted 1:1 with the sample buffer and boiled for 3 min. Silver staining method was used for detecting proteins following gel electrophoresis.

### Total protein quantification

Protein concentrations were measured by the Lowery assay. Bovine serum albumin (BSA) was used as protein standard.

### Size-exclusion high-performance liquid chromatography (SEC-HPLC)

The particle size distribution of various samples (Capto MMC eluate, IAF eluate, cAPI and nAPI) was evaluated using a validated analysis tool of SEC-HPLC. The column used was TSKgel 5000PW of 7.5 mm ID × 60 cm (Tosoh Bioscience GmbH, Griesheim, Germany). The injection volume of the sample on the column was 200 µL. The mobile phase was phosphate-buffered saline (1X PBS, pH 7.4). The flow rate was set at 0.5 ml/min.

### Residual DNA quantification

The residual host cell DNA (HCD) in the samples was measured by an in-house TaqMan Real-Time PCR assay and Applied Biosystems StepOne System (Applied Biosystems, Foster City, California, United States). Total reaction volume was 20 μl, containing 10 μl of 2X Maxima Probe/ROX qPCR Master Mix (Fermentas, Waltham, MA, USA), 0.4 μM of each of the forward and reverse primers, 0.2 μM of TaqMan probe, and 5 μl of DNA template. Five standards with concentrations of 73.42, 734.2, 7342, 73,420, 734,200 (fg/μl) were prepared. The thermal profile of PCR included 10 min at 95 °C for enzyme activation, followed by 40 cycles of 95 °C for 1 min, and 60 °C for 30 s for 40 cycles^42^.

### Enzyme immunoassay for the measurement of host cell protein (HCP)

To define the residual HCP concentration, cAPI and nAPI samples were analyzed using *P. pastoris* HCP ELISA kit (F140, Cygnus technologies, Southport, United States). The standards and samples (25 μl) were added into the microtiter plate wells coated with anti-*P. pastoris* HCP antibodies. Anti-*P. pastoris* : HRP antibodies (100 μl) was then added into each well and the microtiter plate was placed on an orbital plate shaker at 220 rpm for 3 h, at room temperature. The contents of wells were dumped into waste and the plate was firmly taped over an absorbent paper to remove the residual liquid. The wells were washed with 350 µL of the washing solution. Tetramethylbenzidine (TMB) substrate solution (100 μl) was added to each well and the plate was incubated at room temperature for 30 min. Finally, 100 μl of the stop solution was added to each well and the optical density was measured at 450 nm.

### Circular dichroism spectroscopy (CD)

The API samples (cAPI and nAPI) with protein concentration of 0.15 mg/ml were used for the far-ultra violet (UV) CD studies. The CD spectra of 15 mM phosphate buffer alone and APIs plus the buffer were measured using a spectropolarimeter Jasco J-810 (Tokyo, Japan). The baseline far-UV CD spectrum of the buffer was taken away from the spectra containing the protein to yield the true protein CD spectrum (i.e., intrinsic spectrum). The secondary structures were calculated by Spectra Manager for Windows 95/NT, spectra analysis version 1.53.02, JASCO Corporation.

### Quantification of rHBsAg specific activity

Concentration of rHBsAg was measured by antibody sandwich enzyme immunoassay (Pishtazteb 99004, Tehran, Iran). Briefly, the rHBsAg samples were serially diluted by 100, 1000 and 10,000 folds. Ten microliters of each sample and standard was added to each well of a microtiter plate coated with anti-HBsAg polyclonal antibodies, in duplicate. The conjugated enzyme (50 μl) was next added to each well and mixed gently for 15 s. The plate was completely covered and incubated for 30 min, at 37 °C. The content of the plate was removed and the wells were washed with 300 μl washing buffer for 5 times. Chromogen-substrate solution (100 μl) was added into each well and the plate was incubated for 15 min at room temperature in a dark place. The enzymatic reaction was finally stopped using 100 μl of the stop solution. The plate was read at 450 nm. The samples dilutions which fell within the standard curve limits were used for calculation of rHBsAg quantity.

The specific activity of rHBsAg in samples was calculated as below:

$$\begin{aligned} & {\text{Specific activity }}\left( \% \right) \\ & \quad = {\text{rHBsAg }}\left( {\upmu {\text{g}}/{\text{ml}}} \right){\text{ measured by the enzyme immunoassay}}/{\text{total protein }}\left( {\upmu {\text{g}}/{\text{ml}}} \right){\text{ measured by Lowry assay }} \times { 1}00 \\ \end{aligned}$$

### In vitro relative potency assay (IVRP)

The API samples (cAPI and nAPI) were formulated in the presence of aluminum hydroxide (with a final concentration of 0.5 mg/ml). Several dilutions of the formulated samples and the reference vaccine were made in 1X PBS, pH 7.2 and human hepatitis B immunoglobulin (Kedrion Biopharma, Barga (Lucca), Italy) was added to reach 250 mIU in each sample. The samples were incubated overnight at 4 °C and centrifuged at 10,000 *g* for 10 min. The supernatants were collected and the amount of unbound antibody was measured using the antigen sandwich enzyme immunoassay (Pishtazteb 14001, Tehran, Iran). For this purpose, 100 μl of each supernatant was added to each well of a microtiter plate coated with rHBsAg, in triplicate. The plate was completely covered and incubated for 60 min at 37 °C. The content of the plate was removed and the wells were washed with 300 μl of washing buffer for 5 times. The conjugated enzyme (100 μl) was added to each well and the plate was kept for 30 min at 37 °C. The content of the microtiter plate was removed and the wells were washed with 300 μl of washing buffer for 5 times. The chromogen-substrate solution (100 μl) was added into each well and the plate was incubated for 15 min at room temperature in a dark place. The enzymatic reaction was stopped using 100 μl of stop solution and the plate was read at 450 nm. In order to determine the samples potency relative to the reference HBV vaccine, the collected optical density values were transferred to Bioassay Assist 3.0 for parallel line assay (PLA).

**Figure 13:**
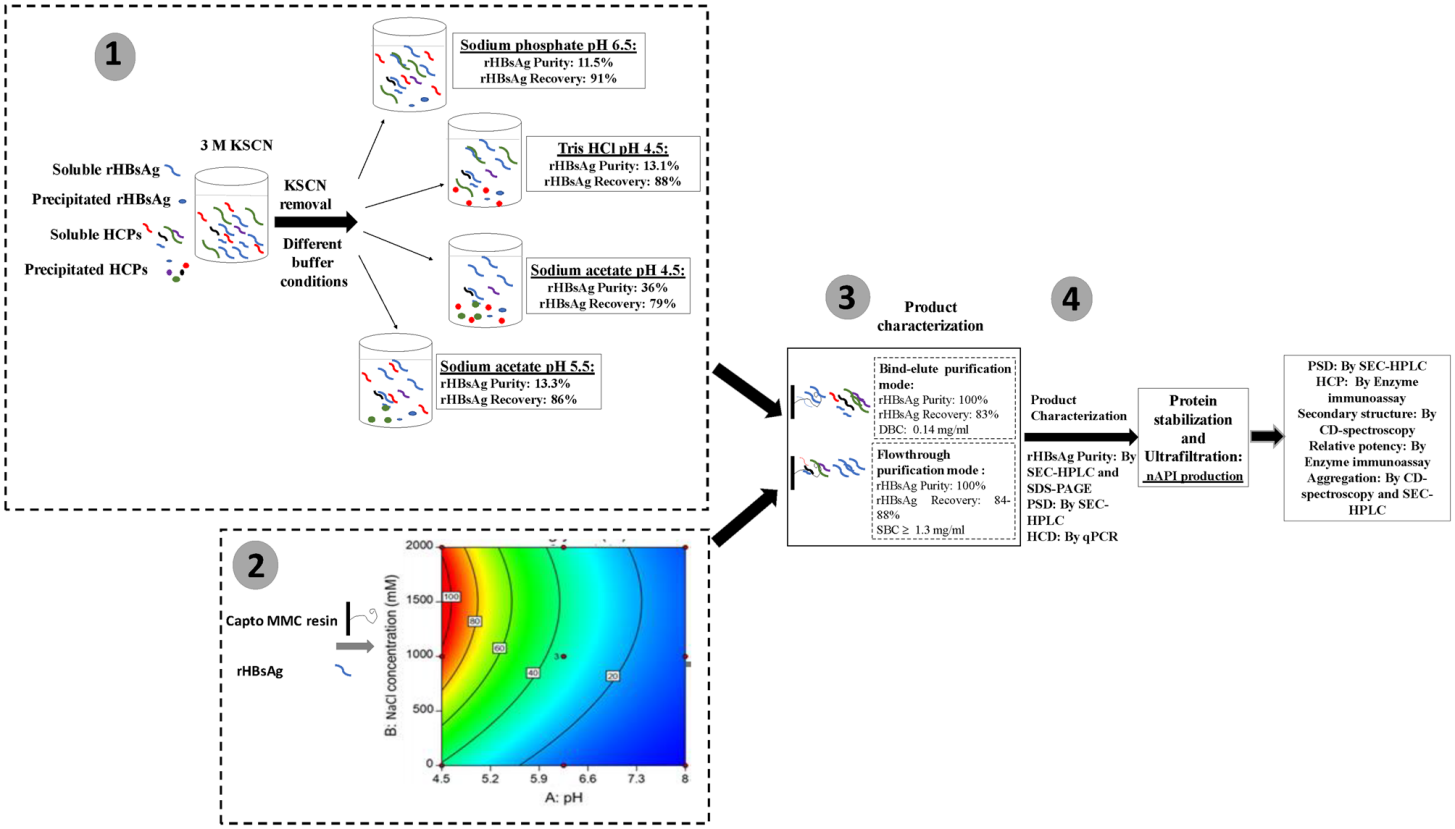
The experimental path.

The experimental path has been depicted in Fig. 13.

## Supplementary Information

Supplementary Information 1.

Supplementary Information 2.

Supplementary Information 3.

Supplementary Information 4.

Supplementary Information 5.

## Data Availability

To request data, please contact MMG.
