# Supplementary material for: Determining buffer conditions for downstream processing of VLP-based recombinant hepatitis B surface antigen using multimodal resins in bind-elute and flow-through purification modes
Source: Sci Rep. 2023 Jul 3;13:10745. doi: 10.1038/s41598-023-37614-y (PMC10318023; doi:10.1038/s41598-023-37614-y)
Supplement: Supplementary file 1 — Supplementary Information 1. [file 41598_2023_37614_MOESM1_ESM.docx]

**Supplementary file S1**

**Binding robustness study**

**Table S1. Full Factorial design (FFD) for rHBsAg binding robustness study using Capto MMC resin in the presence of NaCl**.

| **Run** | **A: pH** | **B: NaCl concentration (mM)** | **Adsorbed rHBsAg (%)** |
| --- | --- | --- | --- |
| 1 | 4.4 | 2000 | 62 |
| 2 | 4.65 | 1800 | 62.7 |
| 3 | 4.9 | 1600 | 62.6 |
| 4 | 4.4 | 1600 | 62.7 |
| 5 | 4.9 | 2000 | 62.51 |
| 6 | 4.65 | 1800 | 62.52 |
| 7 | 4.65 | 1800 | 62.54 |


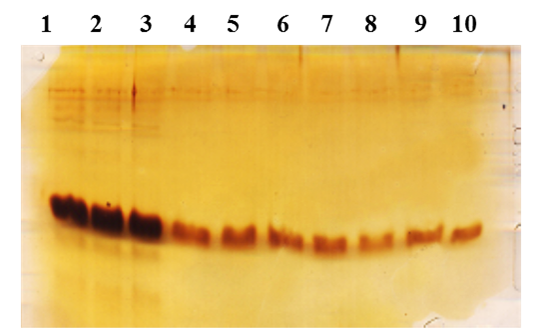


**Figure S1. SDS-PAGE gel for rHBsAg binding robustness study using Capto MMC resin in the presence of NaCl. Lanes 1 to 3 are relevant to the samples before binding at pH 4.9, 4.65, and 4.4, respectively; Lanes 4 to 10 are the supernatant samples collected after performing the FFD test numbers 1 to 7 mentioned in Table S1.**

**Table S2. ANOVA for the rHBsAg binding robustness study using Capto MMC resin in the presence of NaCl.**

| **Source** | **Sum of Squares** | **Df** | **Mean Square** | **F-value** | **p-value** |  |
| --- | --- | --- | --- | --- | --- | --- |
| Model | 0.0000 | 0 |  |  |  |  |
| Residual | 4.640E+07 | 6 | 7.733E+06 |  |  |  |
| Lack of Fit | 4.371E+07 | 4 | 1.093E+07 | 8.11 | 0.1127 | not significant |
| Pure Error | 2.693E+06 | 2 | 1.346E+06 |  |  |  |
| Cor Total | 4.640E+07 | 6 |  |  |  |  |
