# Supplementary material for: Determining buffer conditions for downstream processing of VLP-based recombinant hepatitis B surface antigen using multimodal resins in bind-elute and flow-through purification modes
Source: Sci Rep. 2023 Jul 3;13:10745. doi: 10.1038/s41598-023-37614-y (PMC10318023; doi:10.1038/s41598-023-37614-y)
Supplement: Supplementary file 2 — Supplementary Information 2. [file 41598_2023_37614_MOESM2_ESM.docx]

**Supplementary file S2**

**Elution robustness study**

**Table S2a. Full Factorial design (FFD) rHBsAg elution robustness study using Capto MMC resin in the presence of KSCN.**

| **Run** | **A: pH** | **B: KSCN (M)** | **rHBsAg recovery (%)** | **Purity (%)** |
| --- | --- | --- | --- | --- |
| 1 | 7.8 | 3.3 | 83.2 | 99 |
| 2 | 8 | 3 | 82.5 | 99 |
| 3 | 8.2 | 2.7 | 82.7 | 99 |
| 4 | 7.8 | 2.7 | 81.7 | 99 |
| 5 | 8.2 | 3.3 | 83 | 99 |
| 6 | 8 | 3 | 83 | 99 |
| 7 | 8 | 3 | 81.2 | 99 |


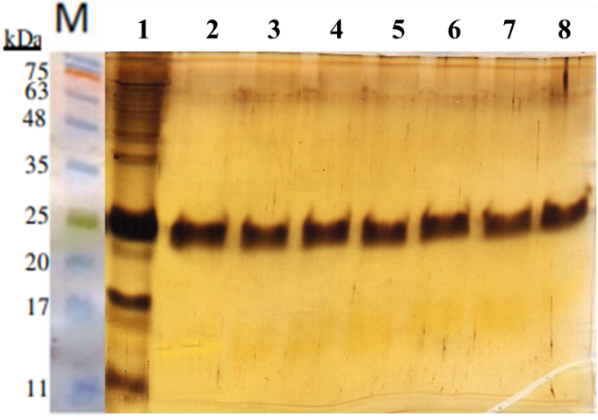


**Figure. SDS-PAGE gel for rHBsAg elution robustness study using Capto MMC resin in the presence of KSCN. 1 is feedstock exposure to Capto MMC resin, and 2-8 are the FFD designed tests.**

**Table S2b. ANOVA table for rHBsAg elution robustness study using Capto MMC resin in the presence of KSCN.**

| **Source** | **Sum of Squares** | **df** | **Mean Square** | **F-value** | **p-value** |  |
| --- | --- | --- | --- | --- | --- | --- |
| Model | 0.0000 | 0 |  |  |  |  |
| Residual | 8.873E+08 | 6 | 1.479E+08 |  |  |  |
| Lack of Fit | 5.860E+08 | 4 | 1.465E+08 | 0.9725 | 0.5638 | not significant |
| Pure Error | 3.013E+08 | 2 | 1.506E+08 |  |  |  |
| Cor Total | 8.873E+08 | 6 |  |  |  |  |
