# Supplementary material for: Determining buffer conditions for downstream processing of VLP-based recombinant hepatitis B surface antigen using multimodal resins in bind-elute and flow-through purification modes
Source: Sci Rep. 2023 Jul 3;13:10745. doi: 10.1038/s41598-023-37614-y (PMC10318023; doi:10.1038/s41598-023-37614-y)
Supplement: Supplementary file 3 — Supplementary Information 3. [file 41598_2023_37614_MOESM3_ESM.docx]

**Supplementary file S3**

**Purification of rHBsAg using unfavorable conditions for binding of rHBsAg on Capto MMC resin**


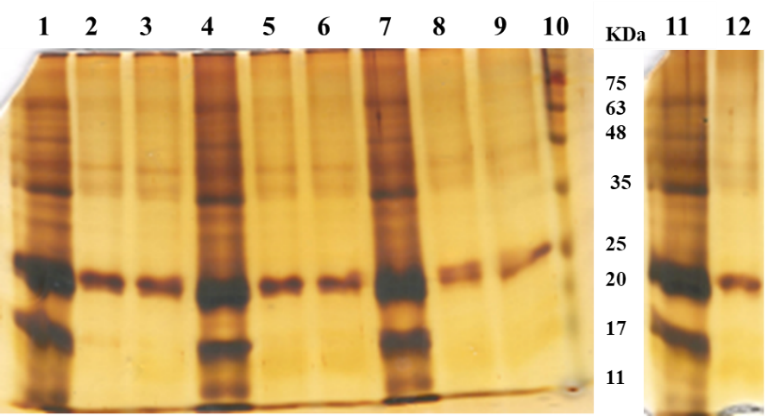


**Figure. First experiment to define unfavorable conditions for binding of rHBsAg on Capto MMC resin. Lanes 1, 4, 7 and 11 are feedstock samples before exposure with Capto MMC resin. Lanes 2, 3, 4, 5, 8, 9 and 12 are respectively the supernatant after exposure with Capto MMC resin at pH 5.5 and NaCl concentrations of 400, 600, 800, 1000, 1200, 1400 and 1600 mM.**

**Description: Figures 7 and 8 in the article show two further repeats for purification of rHBsAg in the flow-through mode at pH 5.5, 600 mM NaCl.**
