# Supplementary material for: Determining buffer conditions for downstream processing of VLP-based recombinant hepatitis B surface antigen using multimodal resins in bind-elute and flow-through purification modes
Source: Sci Rep. 2023 Jul 3;13:10745. doi: 10.1038/s41598-023-37614-y (PMC10318023; doi:10.1038/s41598-023-37614-y)
Supplement: Supplementary file 4 — Supplementary Information 4. [file 41598_2023_37614_MOESM4_ESM.docx]

**Supplementary file S4**

a)

b)


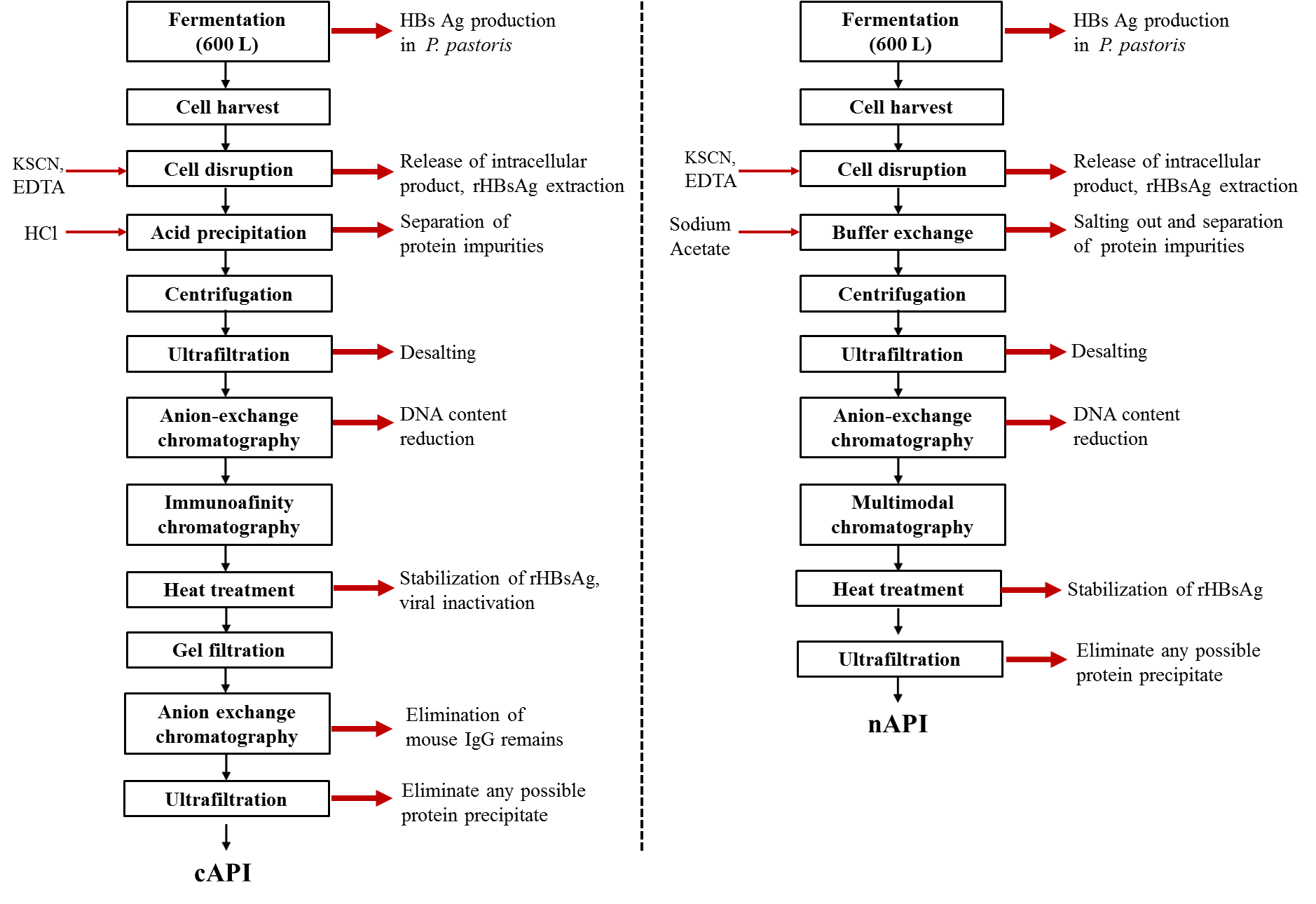


**The conventional rHBsAg API production process (a) versus the newly developed process which is shorter and less expensive downstream process (b).**
