# Supplementary material for: Determining buffer conditions for downstream processing of VLP-based recombinant hepatitis B surface antigen using multimodal resins in bind-elute and flow-through purification modes
Source: Sci Rep. 2023 Jul 3;13:10745. doi: 10.1038/s41598-023-37614-y (PMC10318023; doi:10.1038/s41598-023-37614-y)
Supplement: Supplementary file 5 — Supplementary Information 5. [file 41598_2023_37614_MOESM5_ESM.docx]

**Supplementary file S5**

**Two repeats of rHBsAg SEC-HPLC pattern**


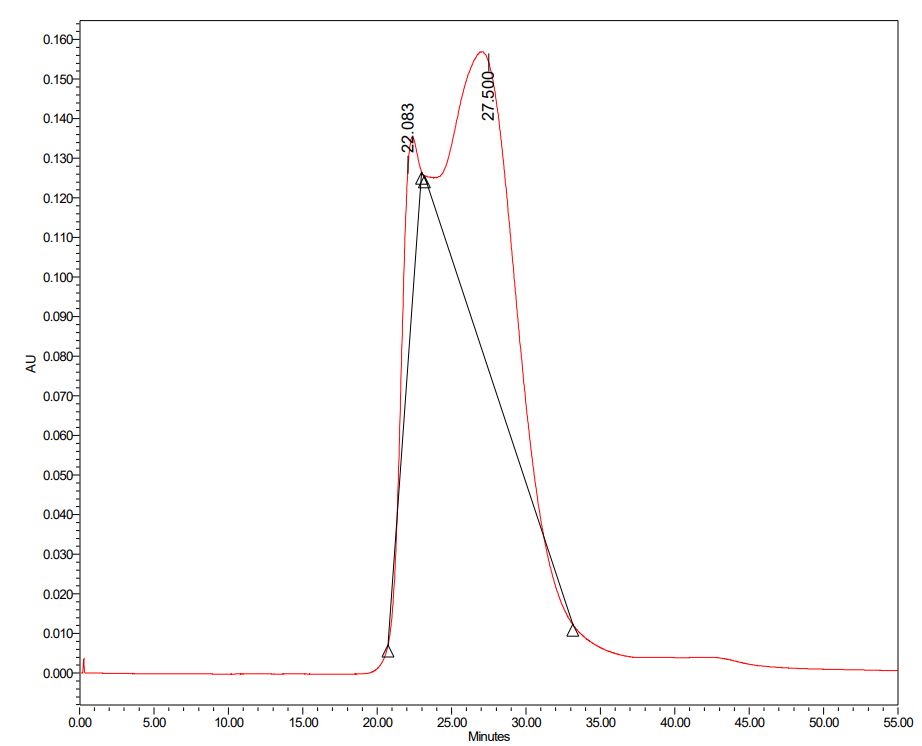


**Figure S4a). SEC-HPLC analysis for particle size distribution of rHBsAg in nAPI.**


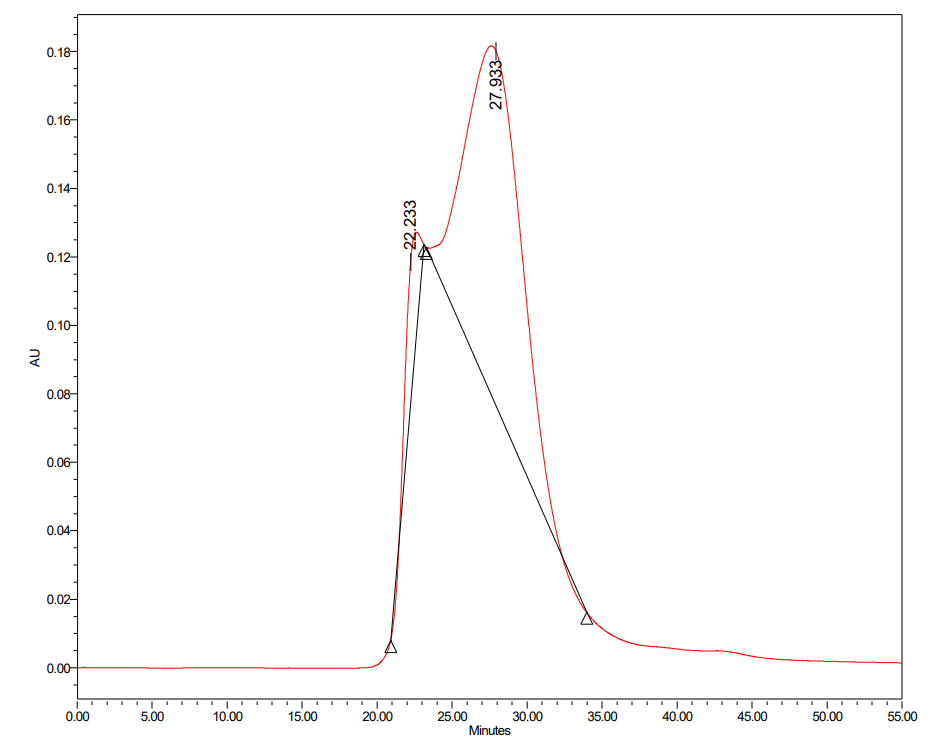


**Figure S5b. SEC-HPLC analysis for particle size distribution of rHBsAg in nAPI (being reported in Figure 9c of the article).**
